# O-GlcNAcylated TCF4 Drives Ferroptosis Resistance and Tumor-Associated Macrophage Infiltration to Promote Colorectal Cancer Liver Metastasis

**DOI:** 10.3390/cancers18193105

**Published:** 2026-09-24

**Authors:** Han Yang, Jinhao Yu, Dong Hou, Jiaxin Lin, Guangyao Chen, Bin Yang, Dongming Lai, Fanghai Han, Hongming Li

**Affiliations:** 1 Department of Laboratory Medicine, The Affiliated Guangdong Second Provincial General Hospital of Jinan University, Guangzhou 510317, China; 2 Department of Gastrointestinal Surgery, Sun Yat-sen Memorial Hospital, Sun Yat-sen University, Guangzhou 510120, China; 3 Department of Gastrointestinal Surgery, Guangdong Provincial Hospital of Traditional Chinese Medicine, Guangzhou 510120, China; 4 Department of Gastrointestinal Surgery, The Affiliated Guangdong Second Provincial General Hospital of Jinan University, Guangzhou 510317, China

**Keywords:** CRLM, O-GlcNAcylation, TCF4, ferroptosis resistance, TAMs

## Abstract

**Background**: Colorectal cancer liver metastasis (CRLM) remains a major cause of mortality in colorectal cancer (CRC), while the upstream molecular mechanisms linking tumor cell adaptation and immune microenvironment remodeling remain poorly understood. **Methods**: By integrating single-cell RNA sequencing, multi-omics analysis, clinical cohorts, and functional experiments, we investigated the role and mechanism of TCF4 post-translational modification in CRLM progression. **Results**: We identified O-GlcNAcylated TCF4 as a critical driver of CRLM. TCF4 was significantly upregulated in CRLM and associated with poor prognosis. Mechanistically, OGT-mediated O-GlcNAcylation at Ser163 promoted TCF4 nuclear localization and enhanced its transcriptional activity. O-GlcNAcylated TCF4 directly activated GPX4 transcription, conferring ferroptosis resistance and preserving the malignant characteristics of CRC cells. Furthermore, TCF4 promoted CSF1 secretion, activating the CSF1/CSF1R axis to facilitate TAM recruitment and M2 macrophage polarization, thereby establishing an immunosuppressive metastatic niche. **Conclusions**: Our study reveals an O-GlcNAcylated TCF4–ferroptosis–immune regulatory axis that coordinates tumor intrinsic adaptation and microenvironmental remodeling during CRLM progression, providing potential therapeutic targets for preventing CRLM.

## Simple Summary

Colorectal cancer is one of the most common cancers worldwide, and its spread to the liver is the leading cause of death in patients with this disease. However, the mechanisms behind this process are not fully understood. In this study, we investigated a protein modification called O-GlcNAcylation and its role in colorectal cancer liver metastasis. We found that a protein called TCF4 is modified by this process, which helps cancer cells survive and spread by protecting them from a type of cell death called ferroptosis. At the same time, this modification helps cancer cells create an immune environment that promotes tumor growth by recruiting immune cells that suppress the body’s natural defense against cancer. Our findings reveal a new regulatory mechanism that drives the spread of colorectal cancer to the liver and suggest potential targets for future therapies to prevent metastasis.

## 1. Introduction

Colorectal cancer (CRC) is one of the leading causes of cancer-related mortality worldwide, and colorectal cancer liver metastasis (CRLM) represents a major clinical challenge limiting long-term patient survival [1,2]. Approximately 40–50% of patients with CRC develop liver metastases during disease progression, which are characterized by early dissemination, high invasiveness, and substantial therapeutic resistance [3,4]. Although multimodal therapeutic strategies, including surgical resection combined with systemic chemotherapy, targeted therapy, and immunotherapy, have been implemented in clinical practice, the five-year survival rate of patients with CRLM remains unsatisfactory due to tumor heterogeneity and the high incidence of postoperative recurrence [5]. Therefore, elucidating the molecular mechanisms underlying the formation of the metastatic liver microenvironment is essential for developing next-generation therapeutic strategies.

The tumor microenvironment (TME), particularly tumor-associated macrophages (TAMs), has been recognized as a critical orchestrator of liver metastasis formation [6]. Accumulating evidence indicates that TAMs participate in the entire metastatic cascade, ranging from the establishment of the pre-metastatic niche to tumor colonization [7]. Tumor cells can “educate” Kupffer cells and recruit peripheral monocytes through the secretion of exosomes and cytokines, thereby establishing an immunosuppressive and pro-angiogenic microenvironment. Although the functions of TAMs in mediating immune escape (e.g., through the PD-1/PD-L2 axis) and vascular remodeling (e.g., through VEGF signaling) have been extensively reported [8,9], the upstream molecular events within tumor cells that initiate this critical recruitment and polarization program remain largely unknown.

Protein post-translational modifications (PTMs), particularly glycosylation modifications, represent fundamental mechanisms regulating proteomic diversity and cellular signal transduction [10,11]. Aberrant glycosylation patterns are hallmarks of malignant tumors and are extensively involved in tumor invasion, metastasis, and immune evasion [12]. Recently, the noncanonical relationship between glycosylation modification and ferroptosis has attracted increasing attention [13]. Although the interplay between ferroptosis resistance and immune microenvironment remodeling has emerged as a potential driver of tumor progression, the mechanisms by which specific glycosylation events bridge tumor metabolic stress responses (ferroptosis) and immune microenvironment reprogramming in CRLM remain poorly understood.

Transcription factor 4 (TCF4), also known as E2-2 or ITF2, is a class I basic helix–loop–helix transcription factor that binds E-box motifs [14]. TCF4/E2-2 has been implicated in CRC cell growth, stemness, and metastasis; however, its post-translational regulation and role in the immune microenvironment of colorectal liver metastasis remain poorly understood [15,16,17]. However, current understanding of TCF4 regulation has primarily focused on its transcriptional activity, whereas whether TCF4 itself is controlled by post-translational modifications and whether such modifications influence its pro-metastatic functions remain largely unexplored. Therefore, this study focused on the glycosylation status of TCF4 and investigated whether TCF4 modification serves as a critical regulatory node linking tumor cell ferroptosis adaptation with immune microenvironment remodeling.

In this study, by integrating single-cell sequencing data and a clinical CRC cohort, we found that TCF4 was a crucial driver in CLRM and conferred ferroptosis resistance to tumor cells. TCF4 expression was elevated in primary CRC tissues from CRLM and was associated with poor prognosis. Mechanistically, TCF4 undergoes O-GlcNAcylation at Ser163, mediated by the OGT glycosyltransferase, which markedly promotes TCF4 nuclear localization. Functionally, O-GlcNAcylated TCF4 not only confers ferroptosis resistance to tumor cells but also promotes M2 macrophage polarization through transcriptional activation of CSF1 secretion, thereby facilitating liver metastasis. This study establishes a novel “O-GlcNAcylated TCF4–ferroptosis–immune” pathogenic axis and provides important theoretical insights and potential therapeutic targets for preventing CRLM.

## 2. Methods

The complete details on the methods and materials are provided in the Supplementary Materials, Supplementary Tables S1 and S2.

### 2.1. Cell Lines and Cell Culture

The uncontaminated colorectal cancer cell lines HCT116 (American Type Culture Collection [ATCC], Manassas, VA, USA; Cat# CCL-247, RRID: CVCL_0291) and DLD1 (ATCC; Cat# CCL-221, RRID: CVCL_0248), and the human monocytic cell line THP-1 (ATCC; Cat# TIB-202, RRID: CVCL_0006), were obtained from the ATCC and maintained in the Tumor Laboratory of the Department of Gastrointestinal Surgery, Sun Yat-sen Memorial Hospital, Sun Yat-sen University. All cell lines were confirmed to be free of mycoplasma contamination by routine testing prior to experimental use. CRC cells were cultured in Dulbecco’s Modified Eagle Medium (DMEM; Gibco, Carlsbad, CA, USA) supplemented with 10% fetal bovine serum (FBS; Invitrogen, Carlsbad, CA, USA). THP-1 cells were cultured in RPMI 1640 medium (1640; Gibco) supplemented with 10% FBS. The culture conditions were set at 37 °C in a humidified atmosphere containing 5% CO_2_. All experiments were carried out during the logarithmic growth phase of the cells.

### 2.2. Statistical Analysis

All statistical analyses were performed using GraphPad Prism 10.0 (GraphPad Software, Inc., San Diego, CA, USA). The data were derived from at least three independent experiments. A *p*-value of less than 0.05 was considered statistically significant. For measurement data, results are presented as the mean ± standard deviation (SD). Statistical significance was analyzed by two-tailed Student’s *t* tests, one-way ANOVA followed by Dunnett’s test, the χ^2^ test, and nonparametric Mann–Whitney U tests as indicated. The overall survival (OS), recurrence-free survival (RFS) and post-progression survival (PPS) of patients were analyzed using the Kaplan–Meier method. Correlations between the groups were analyzed using Spearman’s correlation. The following notation is used to indicate statistical significance: ns, *p* > 0.05; *, *p* < 0.05; **, *p* < 0.01; ***, *p* < 0.001; ****, *p* < 0.0001. Statistical details of each experiment are provided in the relevant figure legends.

## 3. Results

### 3.1. Identification of Ferroptosis-Associated Molecular Subclusters and Characteristic Modules in Colorectal Cancer

To characterize ferroptosis-associated molecular features in colorectal cancer (CRC), we first retrieved 1522 ferroptosis-related genes (FRGs) from the GeneCards database using a relevance score > 1 as the inclusion criterion. Subsequently, using |log2FC| > 1 and FDR < 0.05 as the cutoff criteria, we identified 389 FRGs that exhibited significant expression differences between tumor and normal samples in the TCGA-COAD cohort (Figure 1A and Figure S1A). Univariate Cox regression analysis demonstrated that 25 factors exhibited significant prognostic value and showed correlations with each other (Figure 1B and Figure S1B). In order to accurately identify ferroptosis-associated characteristics in CRC, we applied a non-negative matrix factorization (NMF) algorithm based on the 25 prognostic FRGs to identify characteristic subclusters among patients in the TCGA-COAD cohort. We used the default “brunet” algorithm and determined the optimal number of clusters based on the cophenetic coefficient (Figure 1C and Figure S1C). Two subclusters (C1 and C2) were identified, and Kaplan–Meier survival analysis showed that patients in the C1 subgroup had significantly better prognosis than those in the C2 subgroup (*p* < 0.0001) (Figure 1D). Principal component analysis (PCA), uniform manifold approximation and projection (UMAP), and t-distributed stochastic neighbor embedding (t-SNE) analyses showed that the characteristic subclusters were well-separated and clearly clustered (Figure 1E and Figure S1D,E). Subsequently, we analyzed the 25 prognostic factors and clinical characteristics of patients across different subclusters. As shown in Figure 1F, ferroptosis-associated genes, including *NOTCH3*, *GPX3*, *HAMP*, and *CDKN2A* [18,19,20,21], were significantly upregulated in the C2 subgroup.

To further elucidate the differences in biological functions and potential molecular mechanisms between the C1 and C2 subclusters, we performed comprehensive pathway and functional enrichment analyses based on the transcriptomic data of the two subclusters. KEGG analysis (Figure 1G) and GO enrichment analysis (Figure S1F) consistently demonstrated that extracellular matrix (ECM)–receptor interaction, focal adhesion, and collagen trimer-related pathways were significantly enriched in the C2 subgroup. Gene set enrichment analysis (GSEA) further confirmed that ECM–receptor interaction and cell adhesion molecules (CAMs) were strongly enriched in the C2 subgroup (Figure 1H). Meanwhile, from the perspective of intrinsic metabolism, the peroxisome and oxidative phosphorylation pathways were significantly suppressed in the C2 subgroup. Therefore, we hypothesized that the poor clinical outcomes of the C2 subgroup may be associated with potential ferroptosis resistance. Furthermore, to investigate the potential relationship between the C2 subgroup and tumor microenvironment (TME) heterogeneity, we comprehensively evaluated the microenvironmental characteristics of different subclusters. ESTIMATE algorithm analysis revealed that the stromal score, immune score, and ESTIMATE score were all significantly higher in the C2 subgroup than in the C1 subgroup (Figure 1I). Analysis of immune cell infiltration abundance demonstrated that the infiltration levels of most immune cell populations were significantly increased in the C2 subgroup, with particularly prominent enrichment of classical immunosuppressive components, including myeloid-derived suppressor cells (MDSCs), regulatory T cells, and macrophages (Figure 1J). To more accurately identify characteristic genes closely associated with the C2 subgroup, we constructed a weighted gene co-expression network analysis (WGCNA) based on differentially expressed genes between the two subclusters. First, sample clustering was performed to remove outlier samples, and the soft-thresholding power was selected as β = 12 based on the scale-free network criterion (Figure S1G). Subsequently, the adjacency matrix was transformed into a topological overlap matrix (TOM), and module identification was performed using average linkage hierarchical clustering and dynamic tree cutting methods (with a merge cut height of 0.2 and a minimum module size of 50), resulting in the identification of multiple co-expression modules (Figure 1K). Correlation analysis between co-expression modules and clinical subclusters (Figure 1L) revealed that the yellow module (MEyellow) exhibited the strongest positive correlation with the C2 subgroup (r = 0.55, *p* = 6 × 10^−35^). Further scatter plot analysis of module membership (MM) and gene significance (GS) (Figure 1M) confirmed that genes within the yellow module showed high representativeness and strong internal correlation with the C2 subgroup phenotype (cor = 0.79, *p* = 1.2 × 10^−123^). Therefore, key characteristic genes from the yellow module were selected for subsequent analyses.

### 3.2. Development of a Ferroptosis-Associated Prognostic Risk Model and a Liver Metastasis Diagnostic Model in Colorectal Cancer

To further elucidate the clinical prognostic value of ferroptosis-associated characteristic genes in CRC and explore their potential mechanisms in liver metastasis, we constructed relevant predictive models. First, we constructed a risk score model based on the characteristic genes identified from the previously screened yellow module. The study cohort was randomly divided into an internal training cohort and an internal validation cohort at a ratio of 7:3. In addition, the GSE39582 dataset (*n* = 566) was included as an independent external validation cohort. We then performed LASSO regression analysis (Figure S2A) followed by multivariate Cox proportional hazards regression analysis, and eight optimal characteristic factors were selected based on the minimum partial likelihood deviation to establish a prognostic risk score model (Figure 2A). To comprehensively evaluate the performance of this model, Kaplan–Meier survival analysis was performed. The results demonstrated that patients in the high-risk group had significantly shorter overall survival in the internal training cohort (*p* < 0.001, Figure 2B), internal validation cohort (*p* = 0.005, Figure S2B), and external validation cohort (*p* = 0.003, Figure 2C). Meanwhile, the area under the receiver operating characteristic curve (AUC) values of the model for predicting 1-, 3-, and 5-year survival in the internal training cohort were 0.721, 0.769, and 0.776, respectively (Figure 2D). Together with the consistently stable AUC performance in the internal validation cohort (0.678, 0.708, and 0.732; Figure S2C) and external validation cohort (0.698, 0.668, and 0.678; Figure 2E), as well as the increasing mortality trend revealed by risk factor distribution plots (Figure 2F and Figure S2D,E), these findings strongly confirmed the accuracy and robustness of this model for prognostic prediction in CRC. Furthermore, Cox regression analysis and clinical characteristic stratification analysis revealed that age, overall clinical stage, and the risk score established in this study were independent risk factors affecting the prognosis of CRC patients (Figure 2G and Figure S2F–J). We then developed an integrated nomogram (Figure 2H), which exhibited favorable calibration performance and cumulative risk assessment capability (Figure 2I,J). Furthermore, considering that the ferroptosis-resistant phenotype is frequently associated with malignant progression and distant metastasis, we also focused on liver metastasis: the GSE68468 dataset (190 primary CRC samples and 55 liver metastasis samples) was included for further analysis. Differential expression analysis identified 1604 differentially expressed genes (Figure 2K and Figure S3A). Subsequently, machine learning algorithms were applied for further exploration, and we found that the random forest (RF) algorithm exhibited superior performance compared with the support vector machine (SVM) algorithm in predicting liver metastasis (Figure 2L–N and Figure S3B). Using the RF algorithm, we accurately identified nine core feature genes (*VTN*, *CDH2*, *ADAMTS5*, *APCS*, *PCDHGB6*, *NOX4*, *SNRNP25*, *ITIH3*, and *TCF4*), and subsequently established a liver metastasis-specific diagnostic nomogram based on these genes (Figure 2O,P). This model demonstrated reliable discrimination and a broad range of clinical net benefits in multiple clinical utility evaluations (Figure S3C–E). Notably, the core gene TCF4 was identified not only as a significant high-risk factor in the aforementioned eight-gene prognostic risk model but also as a key feature gene for predicting CRLM in the machine learning analysis. These findings strongly suggest that aberrant TCF4 upregulation may serve as a critical bridge and regulatory hub in the pathological cascade that drives ferroptosis resistance in CRC cells, facilitates metastatic colonization within the liver microenvironment, and ultimately contributes to poor patient outcomes.

### 3.3. Single-Cell Sequencing Reveals Ferroptosis-Associated Malignant Cell Subpopulations and the Regulatory Network of TCF4 in the Colorectal Cancer Liver Metastasis Microenvironment

To better characterize the specific features of ferroptosis-related tumor cells in the colorectal cancer liver metastatic microenvironment, we performed single-cell RNA sequencing (scRNA-seq) analysis. Using the GSE164522 dataset, we carried out dimensionality reduction and clustering of 73,904 cells from the colorectal cancer liver metastasis (CRLM) microenvironment and identified B cells, CD4+ T cells, CD8+ T cells, dendritic cells, fibroblasts, macrophages, malignant cells, mast cells, natural killer cells, plasma cells, and regulatory T cells (Figure 3A,B and Figure S4A). We subsequently subjected the malignant cells to further subclustering (Figure 3C). Based on the 25 previously identified ferroptosis-related prognostic genes, the malignant cells were predominantly classified into two distinct clusters, designated as cluster 1 and cluster 2 (Figure 3D,E and Figure S4B). Compared with cluster 1, cells in cluster 2 exhibited a significantly higher proliferation signature score (Figure 3F) and markedly enhanced tumor stemness (Figure 3G,H). Accordingly, to explore the profound intra-tumoral heterogeneity exclusively within the epithelial malignant cell population, cluster 2 was defined as the ferroptosis-related malignant cell group (FM), whereas cluster 1 was defined as the non-ferroptosis-related malignant cell group (NFM) (Figure 3I). It should be noted that this FM classification is based on a transcriptomically inferred ferroptosis-associated signature. While the elevated proliferation and stemness characteristics reflect an aggressive phenotype, the definitive ferroptosis-resistant biological function driven by core genes within this subpopulation requires further experimental validation. Moreover, TCF4, a core gene identified by the preceding prognostic and diagnostic models, was also significantly upregulated in the FM group (Figure 3J). To further elucidate the intrinsic regulatory mechanisms through which FM cells maintain their ferroptosis-associated malignant phenotype, we performed high-dimensional weighted gene co-expression network analysis (hdWGCNA) on the malignant cell clusters. After determining the optimal soft-thresholding power (Figure S4C) and constructing the gene clustering dendrogram (Figure 3K), module–phenotype correlation analysis showed that the turquoise module exhibited the strongest positive correlation with the FM phenotype (r = 0.65, *p* = 4.6 × 10^−136^) (Figure 3L). Scatterplot analysis further confirmed the strong association between genes in this module and the FM phenotype (r = 0.889, *p* < 0.001; Figure 3M), while both the spatial activation pattern and overall activity of the turquoise module were significantly greater in the FM group than in the NFM group (Figure S4D–E). Kyoto Encyclopedia of Genes and Genomes (KEGG) and Gene Ontology (GO) enrichment analyses of genes within this core module revealed their potential biological functions (Figure 3N and Figure S4F). Notably, gene set enrichment analysis (GSEA) indicated that the O-glycosylation pathway was significantly activated in the FM group (Figure S4G). Quantitative scoring further confirmed that O-glycosylation levels were significantly elevated in the FM group, whereas N-glycosylation levels did not differ significantly between the two groups (Figure 3O,P). In addition, several key metabolism- and glycosylation-related genes displayed marked expression heterogeneity between the FM and NFM groups (Figure S4H). We also performed pseudotime trajectory analysis to investigate cell-fate dynamics during the transition of malignant cells toward the FM phenotype (Figure S4I,J). The analysis showed that FM and NFM cells occupied distinct branches along the evolutionary trajectory and that O-glycosylation scores increased dynamically and markedly during cell-fate progression (Figure S4K,L). Finally, to investigate the functional interactions of FM cells within the tumor microenvironment (TME), we used CellChat to evaluate the intercellular communication networks between FM cells and other cell types. The results showed that FM cells communicated not only with other malignant cell populations but also through extensive and complex interaction networks with macrophages, fibroblasts, and various lymphocyte subsets in the microenvironment (Figure 3Q). These findings strongly suggest that specific microenvironmental interaction networks may exert critical synergistic effects in mediating ferroptosis resistance and metabolic reprogramming in CRLM tumor cells. Collectively, these single-cell findings are highly consistent with the results of our preceding models and provide further evidence that TCF4 may act as a central driver of liver metastatic progression and tumor aggressiveness by inducing ferroptosis resistance in CRC cells and remodeling the tumor immune microenvironment.

### 3.4. TCF4 Promotes Ferroptosis Resistance and Facilitates Colorectal Cancer Liver Metastasis

Based on our previous findings, we further validated the upregulated expression pattern of TCF4 in CRC tissues using the GEO dataset GSE271719 (Figure 4A). Subsequently, immunohistochemistry (IHC) staining was performed to further confirm the expression pattern of the TCF4 protein in CRC tissues. The results demonstrated that TCF4 expression was markedly increased in primary CRC tissue with liver metastasis compared with normal tissues (Figure 4B). Kaplan–Meier survival analysis showed that high TCF4 expression was significantly associated with poor overall survival, relapse-free survival, and post-progression survival in CRC patients (Figure 4C and Figure S5A,B). These findings strongly suggest that TCF4 functions as an oncogenic factor during CRC progression. To investigate the biological function of TCF4 in regulating ferroptosis, we established CRC cell models with TCF4 knockdown (Figure S5C) and TCF4 overexpression (Figure S5D). We found that TCF4 knockdown enhanced erastin-induced ferroptosis (Figure 4D and Figure S5E). This effect was reversed by treatment with the ferroptosis inhibitors ferrostatin-1 and liproxstatin-1, whereas treatment with an apoptosis inhibitor (Z-VAD-FMK), a necroptosis inhibitor (necrosulfonamide), and the autophagy inhibitor 3-methyladenine did not exert a similar rescue effect (Figure 4E,F and Figure S5F,G). Conversely, CRC cells overexpressing TCF4 exhibited strong resistance to erastin-induced ferroptosis (Figure 4G,H and Figure S5H–J). Furthermore, TCF4 knockdown increased the production of MDA and 4-HNE in CRC cells, whereas TCF4 overexpression reduced the production of MDA and 4-HNE (Figure 4I,J and Figure S5K,L). In the xenograft tumor model, the growth inhibition caused by TCF4 knockdown was abolished by liproxstatin-1 treatment (Figure 4K–N). The liver metastasis model was established by tail vein injection of HCT116 cells. In this model, TCF4 knockdown significantly reduced the formation of liver metastatic lesions, whereas this protective effect was markedly reversed following liproxstatin-1 treatment (Figure 4O,P). Collectively, these findings demonstrate that TCF4 not only promotes CRC tumor growth and liver metastasis both in vitro and in vivo, but also plays a central regulatory role in tumor progression by inducing ferroptosis resistance.

### 3.5. O-GlcNAcylation of TCF4 at Ser163 Mediates Ferroptosis Resistance in Colorectal Cancer Cells Through Regulation of Nuclear Translocation

Based on our single-cell transcriptomic analysis, the FM tumor cell subpopulation exhibited significantly elevated global O-glycosylation pathway activity. It is crucial to distinguish this general extracellular and intracellular O-glycosylation inferred from transcriptomics from specific intracellular regulatory modifications. Because O-GlcNAcylation serves as the predominant intracellular O-glycosylation modification regulating transcription factor activity, we hypothesized that the highly expressed TCF4 might be specifically regulated by O-GlcNAcylation at the protein level in CRC cells. Using LC-MS/MS, we identified the O-GlcNAcylation sites of TCF4 in HCT116 cells (Figure 5A). Considering that OGT is a key enzyme responsible for O-GlcNAcylation, we first established OGT knockdown cells in HCT116 and DLD1 cells and found that OGT knockdown affected the O-GlcNAcylation level of TCF4 (Figure 5B and Figure S6A,B). Moreover, the S163A mutation significantly reduced TCF4 O-GlcNAcylation (Figure 5C). Previous studies have demonstrated that transcription factors may enhance nuclear localization through O-GlcNAcylation to activate downstream transcriptional programs [22]. Therefore, we sought to investigate whether O-GlcNAcylation of TCF4 affects its nuclear–cytoplasmic distribution. The results showed that OGT knockdown significantly reduced the nuclear expression level of wild-type TCF4 (TCF4-WT), suggesting that O-GlcNAc modification is essential for TCF4 nuclear localization. In addition, the nuclear expression of the TCF4-S163A mutant was markedly reduced and was not affected by OGT knockdown. These findings further confirmed that O-GlcNAc modification at the Ser163 site serves as a molecular switch regulating TCF4 nuclear translocation and function (Figure 5D). Subsequently, we investigated the role of TCF4 O-GlcNAcylation in CRC cells. Notably, the S163A mutant cells exhibited increased sensitivity to erastin-induced ferroptosis, indicated by cell death (Figure 5E), cell viability (Figure 5F), and the levels of lipid peroxidation products MDA (Figure 5G) and 4-HNE (Figure 5H). To exclude the influence of endogenous TCF4, we knocked down TCF4 using the most efficient siRNA. Consistent with our previous results, TCF4 knockdown reduced nuclear localization and increased erastin-induced ferroptosis, which was rescued by TCF4-WT but not by TCF4-S163A (Figure S6C–G). Furthermore, in TCF4-knockdown CRC cells reconstituted with TCF4-WT or TCF4-S163A, the S163A mutant exhibited greater sensitivity to erastin-induced ferroptosis than TCF4-WT, and this effect was independent of OGT knockdown (Figure S6H–K). Collectively, these results demonstrate that O-GlcNAc modification of TCF4 at Ser163 promotes its nuclear translocation and plays a critical role in regulating ferroptosis resistance in CRC cells.

### 3.6. TCF4 Mediates Ferroptosis Resistance in Colorectal Cancer Cells Through Direct Transcriptional Activation of GPX4

To elucidate the molecular mechanisms underlying TCF4-mediated effects, we performed RNA-seq analysis on CRC cells transfected with TCF4 siRNA or control siNC. In CRC cells, mRNA-seq data showed that TCF4 knockdown significantly reduced GPX4 mRNA levels (Figure 5I). KEGG analysis, GO analysis, and GSEA further demonstrated that TCF4 knockdown affected ferroptosis-related pathways and molecular functions in CRC cells (Figure 5J,K and Figure S7A). Analysis of the GEO database further confirmed the high expression of GPX4 in tumor tissues (Figure S7B). Kaplan–Meier analysis showed that high GPX4 expression was significantly associated with poorer overall survival in CRC patients (Figure S7C). We hypothesized that TCF4, as a transcription factor, enhances GPX4 transcription, leading to increased GPX4 protein expression, which is consistent with our RNA-seq results. Therefore, we compared the GPX4 promoter sequence with the TCF4 binding motifs in the JASPAR database and predicted two potential TCF4 binding sites within the GPX4 promoter (Figure 5L,M). Subsequently, we used a dual-luciferase reporter assay to validate these two potential binding sites. As expected, TCF4 overexpression significantly increased GPX4 promoter activity in CRC cells. However, this effect was abolished after mutation of site 1, but not site 2 (Figure 5N–P), indicating that site 1 is required for TCF4-mediated regulation of GPX4. Furthermore, chromatin immunoprecipitation (ChIP) analysis showed that TCF4 overexpression significantly increased TCF4 enrichment at site 1 of the GPX4 promoter in CRC cells, whereas no such increase was observed at site 2 (Figure 5Q,R). Then, we investigated the effect of TCF4 on GPX4 expression changes. Following TCF4 knockdown in CRC cells, GPX4 expression levels were significantly decreased in HCT116 and DLD1 cells (Figure S7D,E). Conversely, the results were reversed when CRC cells were treated with TCF4 overexpression (Figure S7F,G). In addition, we found that knockdown of OGT in CRC cells markedly suppressed GPX4 mRNA levels (Figure S7H,I). Moreover, in TCF4-knockdown CRC cells, reconstitution with TCF4-S163A failed to restore GPX4 mRNA levels, indicating that O-GlcNAcylation of TCF4 at Ser163 enhances GPX4 transcription (Figure S7J,K). To further investigate whether GPX4 is involved in the role of TCF4 in negatively regulating ferroptosis, we constructed GPX4 RNA interference CRC cells (Figure S7L). The results showed that the TCF4-mediated cell viability effect (Figure 5S and Figure S7M,N) and the production levels of lipid peroxidation products MDA (Figure 5T) and 4-HNE (Figure 5U) were abolished by GPX4 knockdown. RSL3 induces ferroptosis by directly binding to GPX4 and blocking its enzymatic activity. We found that neither OGT knockdown nor TCF4 knockdown increased the sensitivity of CRC cells to RSL3-induced ferroptosis (Figure S7O–R). In summary, these results demonstrate that TCF4 specifically regulates ferroptosis in a GPX4-dependent manner, representing a core molecular mechanism by which CRC cells evade ferroptosis and promote tumor progression.

### 3.7. TCF4 Promotes Macrophage Recruitment and M2-Like Polarization in Colorectal Cancer

Tumor-associated macrophages (TAMs) are important components of the tumor microenvironment and promote tumor growth and invasion. Based on our previous multi-omics analysis results, we further analyzed the correlation between TCF4 expression and immune cells in the TCGA-COAD cohort, and the results showed that TCF4 expression was significantly correlated with macrophage infiltration (Figure S8A). Next, we compared the expression characteristics of CD68 between normal colorectal tissues and colorectal cancer samples. The results showed that the infiltration density of CD68+ cells in metastatic tumors was significantly higher than that in primary lesions, indicating stronger TAM infiltration in the liver metastatic microenvironment (Figure 6A). To further clarify the composition of different TAM phenotypes in the microenvironments of CRLM (−) and CRLM (+) patients, we used IHC staining of tissue samples with CD86 to identify M1 TAMs and CD206 to identify M2 TAMs (Figure 6B). Quantitative analysis showed that more M2 TAMs and fewer M1 TAMs were detected in LM (+) patient samples, and the M2/M1 ratio was significantly increased (Figure 6C–E). In addition, we evaluated the expression of M1 and M2 macrophage markers in tumor samples. The results showed that the expression of M1 macrophage markers, including *TNFa*, *iNOS*, and *CD86*, was decreased in liver metastases (Figure 6F). In contrast, the expression of M2 macrophage markers, including *CD206*, *VEGF*, and *CD163*, was significantly increased in LM (+) samples (Figure 6G). More importantly, Spearman correlation analysis showed that TCF4 expression was correlated with TAM numbers and the expression of M2 macrophage markers in CRC samples (Figure 6H–K). Given that TCF4 expression levels were significantly associated with TAM infiltration density and M2 macrophage markers in CRC patient tissues, we further investigated the function of TCF4 in regulating macrophage recruitment and polarization. We established a co-culture system of CRC cells and THP-1-derived macrophages to determine whether TCF4 mediates crosstalk between them (Figure 6L). Transwell assays showed that the migration efficiency of THP-1-induced macrophages was affected by co-culture with TCF4-knockdown and TCF4-overexpressing CRC cells (Figure 6M–P). When macrophages were co-cultured with CRC cells overexpressing TCF4, the expression of M1 markers (such as *TNFa*, *iNOS*, and *CD86*) was significantly decreased (Figure 6Q), whereas M2 markers (such as *CD206*, *VEGF*, and *CD163*) were significantly upregulated (Figure 6R). Conversely, when macrophages were co-cultured with TCF4-deficient CRC cells, the aforementioned effect of inducing macrophage M2 polarization was significantly weakened (Figure 6S,T). Moreover, in TCF4-knockdown CRC cells, reconstitution with TCF4-S163A failed to restore the induction of macrophage M2 polarization, indicating that O-GlcNAcylation of TCF4 at Ser163 is required for CRC cell-mediated TAM recruitment and M2 polarization (Figure S9A–D). In addition, IHC analysis in the liver metastasis model further confirmed that the expression of the M2 macrophage marker CD206 was significantly decreased in liver metastatic lesions of mice in the TCF4 knockdown group (Figure 6U). Collectively, these findings indicate that TCF4 regulates the crosstalk between CRC cells and macrophages by recruitment and M2-like polarization.

### 3.8. TCF4 Induces TAM Recruitment and Polarization Through Regulation of the CSF1/CSF1R Axis

To further elucidate the molecular mechanisms by which TCF4 regulates TAM recruitment and polarization, we measured the expression levels of multiple chemokines in various CRC cells using the ELISA (Figure 7A–H and Figure S10A–H). The results showed that CSF1 was the most significantly altered under the regulation of TCF4. As an important immune regulatory factor, CSF1 recruits monocytes, memory T cells, and dendritic cells through the CSF1/CSF1R signaling axis and plays a central role in tumor progression. We first evaluated the effects of different TCF4 expression levels on CSF1. qPCR results showed that TCF4 knockdown and overexpression significantly decreased and increased CSF1 mRNA expression levels, respectively, in HCT116 and DLD1 cells (Figure 7I–L). Moreover, in TCF4-knockdown CRC cells, reconstitution with TCF4-S163A failed to restore both CSF1 mRNA levels and protein concentrations, indicating that O-GlcNAcylation of TCF4 at Ser163 enhances CSF1 expression. (Figure S10I–K). IHC analysis in tissues from the liver metastasis model further confirmed that TCF4 knockdown reduced the expression of CSF1 and its receptor CSF1R (Figure 7M). Consistent with the above in vitro and in vivo results, immunohistochemical analysis of clinical samples showed that the expression levels of CSF1 and CSF1R were significantly higher in tumor tissues from CRLM (+) patients than in those from CRLM (−) patients or normal colorectal tissues (Figure 7N). To clarify the specific molecular mechanism by which TCF4 regulates CSF1, we compared the CSF1 promoter sequence with the TCF4 binding motifs in the JASPAR database and predicted two potential TCF4 binding sites within the CSF1 promoter (Figure 7O). Subsequently, we used dual-luciferase reporter assays to validate these two potential binding sites (Figure 7P). As expected, TCF4 overexpression significantly increased CSF1 promoter activity in CRC cells. However, this effect was abolished after mutation of site 1, but not site 2 (Figure 7Q and Figure S10L), indicating that site 1 is required for TCF4-mediated regulation of CSF1. Consistently, ChIP analysis showed that TCF4 overexpression significantly increased TCF4 enrichment at site 1 of the CSF1 promoter in CRC cells, whereas no such increase was observed at site 2 (Figure 7R and Figure S10M). Moreover, the addition of a CSF1-neutralizing antibody to the co-culture medium of macrophages and TCF4-overexpressing CRC cells significantly attenuated TCF4-induced macrophage M2 polarization, indicating that CRC cell-secreted CSF1 is required for TAM recruitment and M2 polarization (Figure S10N–Q). Collectively, these data demonstrate that O-GlcNAcylation of TCF4 at Ser163 enhances its binding to and activation of the CSF1 promoter, thereby precisely regulating CSF1 expression at the transcriptional level. This activates the CSF1/CSF1R signaling axis, promotes TAM infiltration, and reshapes the immunosuppressive microenvironment, ultimately facilitating colorectal cancer liver metastasis progression.

## 4. Discussion

The dynamic interactions between colorectal cancer (CRC) cells and the tumor microenvironment (TME) serve as a central driving force for liver metastasis [23,24]. Although previous studies have demonstrated that the TME reshapes metabolic and immune landscapes through various extracellular signals [25,26], the underlying “molecular drivers” initiating these signaling cascades remain incompletely understood. In this study, we identified aberrant protein glycosylation, a key hallmark of malignant transformation [27,28], as a critical upstream driver of colorectal cancer liver metastasis (CRLM). Our findings fill a conceptual gap between PTM and TME remodeling, revealing TCF4 glycosylation as a critical molecular switch that coordinately regulates ferroptosis resistance within tumor cells and immune evasion in TME.

Previous studies have implicated TCF4/E2-2 in the maintenance of the stemness and metastatic properties of colorectal cancer cells [15]. Although phosphorylation-dependent regulation of TCF4/E2-2 has been reported [29], the PTM mechanisms governing TCF4/E2-2 in CRC remain poorly understood. This study provides the first evidence that TCF4 undergoes OGT-mediated O-GlcNAc modification at the Ser163 site in CRC cells, greatly expanding the regulatory mechanism of TCF4. Notably, the regulation of ferroptosis by glycosylation exhibits substantial heterogeneity among different cancer types: the USP8/OGT axis suppresses ferroptosis sensitivity in hepatocellular carcinoma by stabilizing SLC7A11 [30], whereas ZEB1 glycosylation induces lipid peroxidation-driven ferroptosis in pancreatic cancer [31]. In contrast, our study reveals a unique “pro-metastatic” role of ferroptosis in CRC: O-GlcNAcylated TCF4 transcriptionally activates GPX4 to precisely regulate intracellular ferroptosis resistance, thereby paradoxically maintaining tumor cell stemness and invasive capacity. We confirmed this mechanism through luciferase reporter assays and rescue experiments. More importantly, mutation of the Ser163 site in TCF4 abolished this effect, confirming that O-GlcNAcylation-mediated ferroptosis resistance represents a driving event. Beyond conferring intrinsic survival advantages, this study further revealed that O-GlcNAcylated TCF4 promotes CSF1 release through transcriptional activation. Our findings suggest that the TCF4–CSF1 axis promotes macrophage recruitment and a shift toward an M2-like phenotype. However, the conventional M1/M2 framework represents a simplified classification. Further single-cell analyses are required to define the specific macrophage states regulated by the TCF4–CSF1 axis. Although targeting the CSF1/CSF1R axis has shown therapeutic potential in multiple solid tumors [32,33], our findings demonstrate that this secretory phenotype is directly driven by an O-GlcNAcylation transcription factor in CRC, providing a novel mechanistic explanation for the establishment of the liver metastatic microenvironment. It is important to note certain limitations in our study. Firstly, the liver-metastasis diagnostic model constructed via machine learning was based solely on the GSE68468 dataset. Due to the limited availability of external cohorts with matched primary and metastatic transcriptomic data, this model lacks independent external validation. Therefore, the diagnostic performance and potential clinical utility of this nine-gene signature should be interpreted with caution and require further validation in large-scale, prospective, independent cohorts. Secondly, our clinical analyses measured total TCF4 expression rather than TCF4 O-GlcNAcylation. Direct O-GlcNAcylation of TCF4 at Ser163 was demonstrated in CRC cell lines, and whether this modification is elevated in human CRLM tissues requires further validation. Finally, pulmonary and other-organ metastatic burdens were not systematically evaluated in our study. Future studies using orthotopic, intrasplenic, or intraportal models will be required to explore the role of TCF4 in specific CRC liver metastasis.

In summary, this study establishes a complex dual-regulatory model: O-GlcNAcylated TCF4 promotes tumor cell adaptability by suppressing ferroptosis via the GPX4 pathway, while concurrently fostering an immunosuppressive microenvironment through CSF-1-mediated recruitment of TAMs. These findings not only deepen our understanding of PTM-driven organ-specific metastasis but also provide a theoretical basis for targeting the O-GlcNAcylated TCF4 and CSF1/CSF1R axis as a therapeutic strategy in CRLM.

## 5. Conclusions

This study identifies O-GlcNAcylated TCF4 as a critical molecular driver of CRLM and reveals its dual regulatory functions in tumor cell adaptation and immune microenvironment remodeling. We demonstrate that OGT-mediated O-GlcNAcylation of TCF4 at Ser163 enhances TCF4 nuclear localization and transcriptional activity, thereby activating GPX4 expression and conferring ferroptosis resistance to CRC cells. In parallel, O-GlcNAcylated TCF4 promotes CSF1 transcription and secretion, activating the CSF1/CSF1R signaling axis to induce TAM recruitment and M2 polarization, ultimately generating an immunosuppressive metastatic niche. Targeting the O-GlcNAcylated TCF4–GPX4 ferroptosis pathway or the TCF4–CSF1/CSF1R immune regulatory axis may provide promising therapeutic strategies for CRLM.

## Figures and Tables

**Figure 1:**
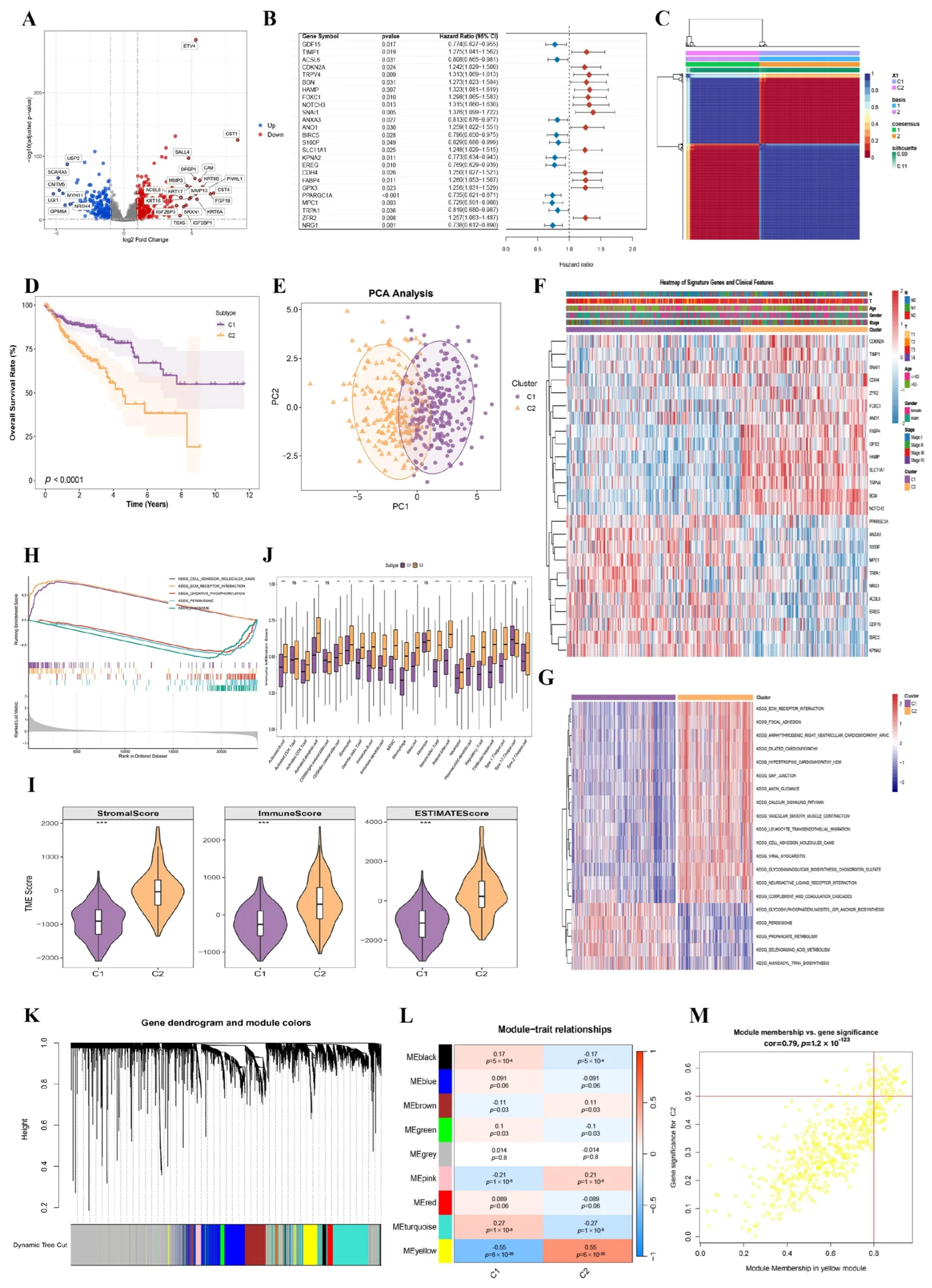
Identification of ferroptosis-associated molecular subclusters and characteristic modules in colorectal cancer. (**A**) Volcano plot showing the differentially expressed ferroptosis-related genes (FRGs) between tumor and normal samples in the TCGA−COAD cohort. (**B**) Forest plot showing the univariate Cox regression analysis of 25 FRGs with significant prognostic value. (**C**) Non−negative matrix factorization (NMF) consensus clustering matrix based on 25 prognostic FRGs (optimal number of clusters k = 2). (**D**) Kaplan−Meier overall survival (OS) curves for patients in the C1 and C2 subclusters. (**E**) Principal component analysis (PCA) plot of the C1 and C2 subclusters. (**F**) Heatmap showing the expression distribution of 25 prognostic FRGs and related clinical features of patients in the C1 and C2 subclusters. (**G**) Heatmap showing the KEGG pathway enrichment analysis between the C1 and C2 subclusters. (**H**) Gene set enrichment analysis (GSEA) revealing the differentially enriched signaling pathways between the C1 and C2 subclusters. (**I**) Comparison of stromal scores, immune scores, and ESTIMATE scores between the C1 and C2 subclusters evaluated by the ESTIMATE algorithm. (**J**) Differential analysis of the infiltrating abundance of various immune cells in the C1 and C2 subclusters. (**K**) Gene clustering dendrogram and module color assignment in weighted gene co−expression network analysis (WGCNA). (**L**) Heatmap showing the correlation between WGCNA co−expression modules and clinical subclusters (C1, C2). (**M**) Scatter plot showing the high positive correlation between module membership (MM) of genes in the yellow module and gene significance (GS) for the C2 subcluster. The statistical difference was assessed through a two-sided Mann–Whitney U test in (**I**,**J**). *, *p* < 0.05; **, *p* < 0.01; ***, *p* < 0.001.

**Figure 2:**
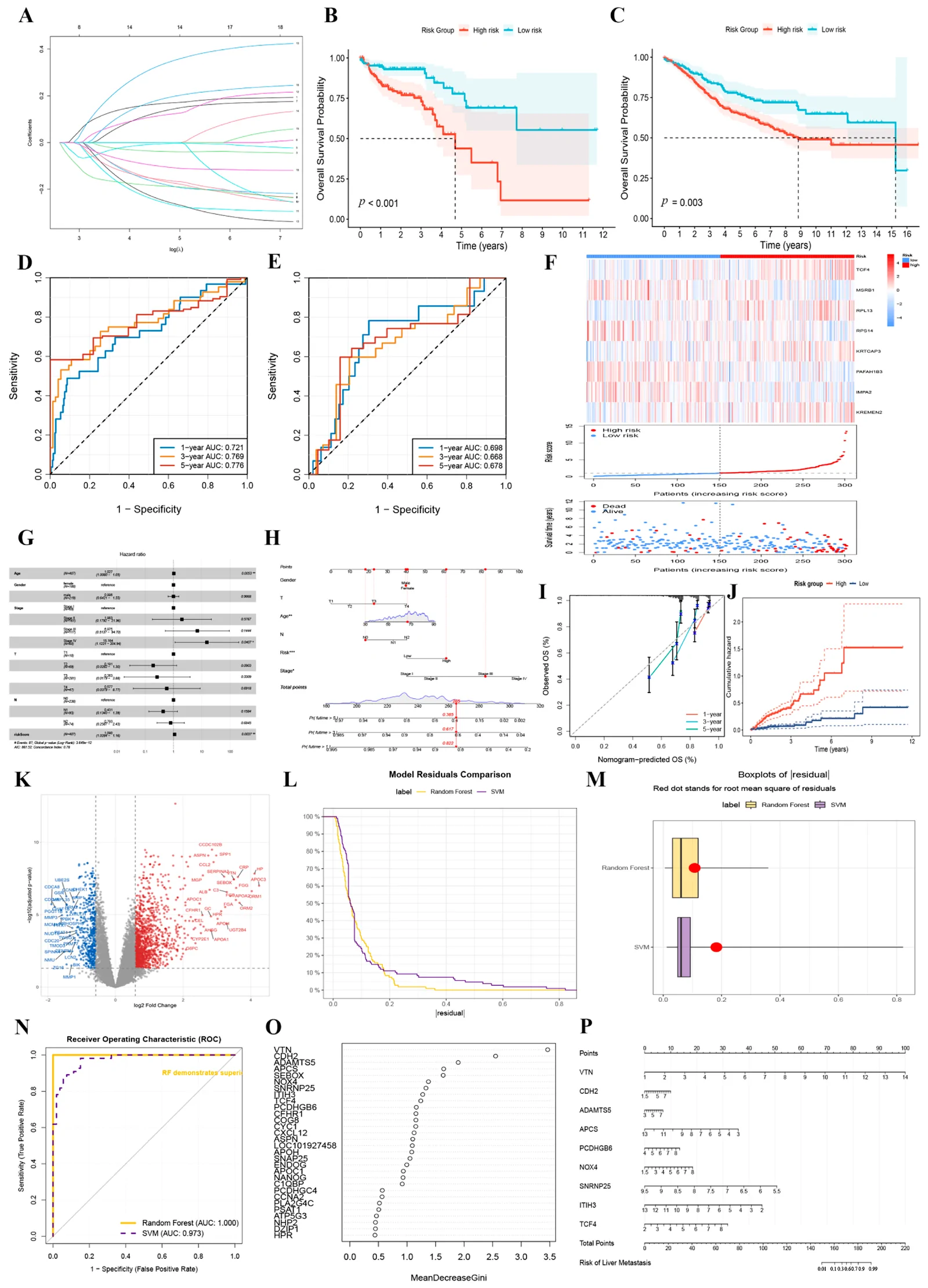
Development of a ferroptosis-associated prognostic risk model and a liver metastasis diagnostic model in colorectal cancer. (**A**) Coefficient distribution of prognostic signature genes in LASSO regression analysis. (**B**) Kaplan–Meier overall survival (OS) curves for patients in the high–and low–risk groups in the internal training set. (**C**) Kaplan–Meier overall survival curves for patients in the high– and low–risk groups in the external validation set (GSE39582). (**D**) Time-dependent ROC curves for predicting 1–, 3–, and 5–year OS in the internal training set. (**E**) Time-dependent ROC curves for predicting 1–, 3–, and 5–year OS in the external validation set. (**F**) Distribution of risk scores, patient survival status, and expression heatmap of the eight model genes in the internal training set. (**G**) Forest plot of the multivariate Cox regression analysis integrating clinical features such as age, clinical stage, and risk score. (**H**) Comprehensive nomogram combining the risk score and clinical features to predict the OS of CRC patients. (**I**) Calibration curve of the comprehensive nomogram for predicting OS. (**J**) Cumulative hazard evaluation curves for patients in the high- and low-risk groups. (**K**) Volcano plot of differentially expressed genes between normal primary CRC and CRC with liver metastasis (LM). (**L**) Residual distribution comparison between the random forest (RF) and support vector machine (SVM) models. (**M**) Box plot of the root mean square residuals for the RF and SVM models. (**N**) ROC curves comparing the performance of the RF and SVM models in predicting CRLM. (**O**) Scatter plot ranking the importance of signature genes screened based on the mean decrease in Gini in the RF model. (**P**) Diagnostic nomogram constructed based on nine core signature genes for predicting CRLM. * *p* < 0.05, ** *p* < 0.01, *** *p* < 0.001.

**Figure 3:**
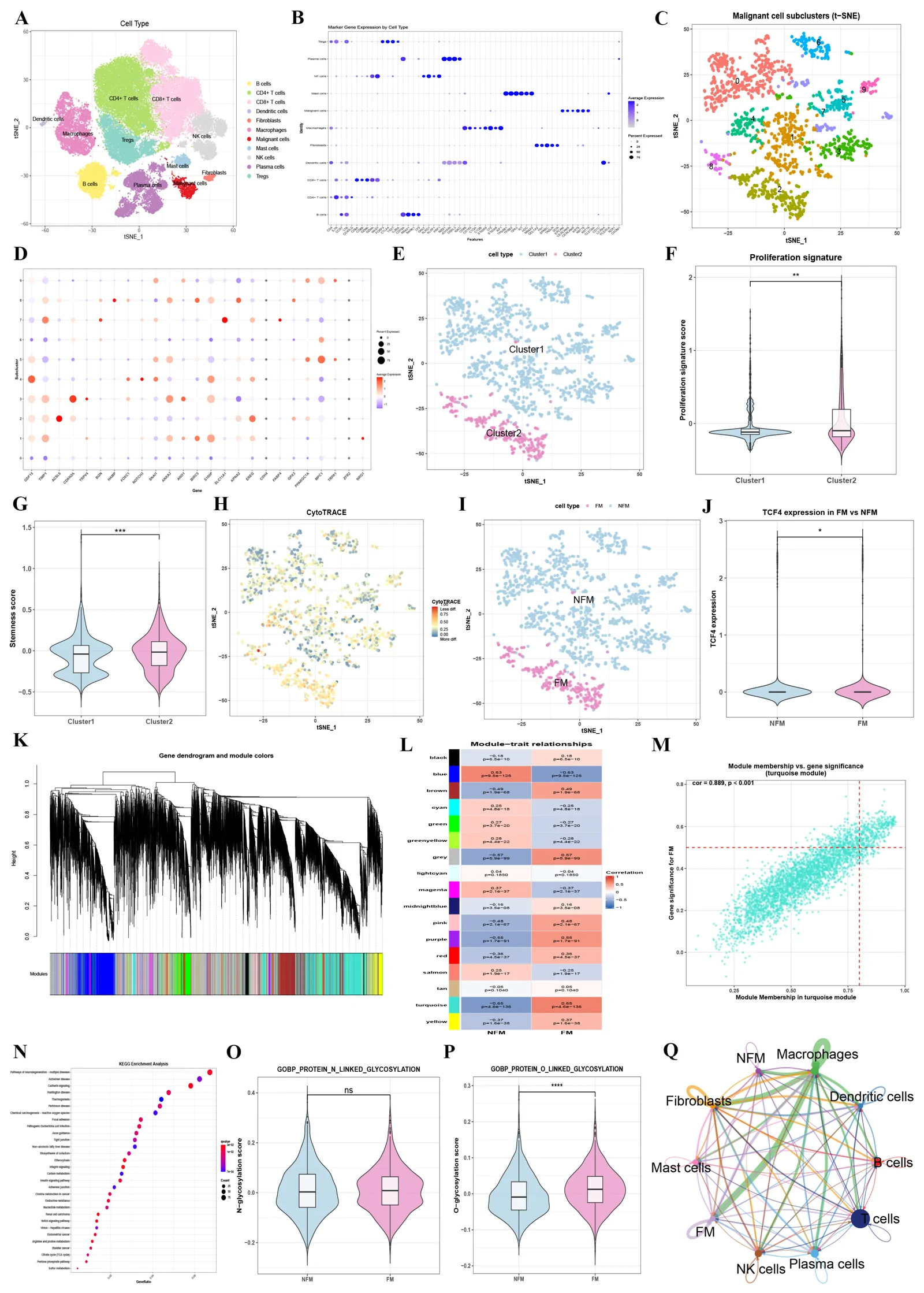
Single-cell sequencing reveals ferroptosis-associated malignant cell subpopulations and the regulatory network of TCF4 in the colorectal cancer liver metastasis microenvironment. (**A**) t-SNE plot for dimensional reduction and clustering of cell types in the CRLM microenvironment based on the GSE164522 dataset. (**B**) Bubble plot showing the expression levels of marker genes across different cell types. (**C**) t-SNE plot for the subdivision of malignant cell subclusters. (**D**) Bubble plot of the expression of 25 ferroptosis-related prognostic signature genes in malignant subclusters. (**E**) t-SNE plot classifying malignant cells into Cluster 1 and Cluster 2 based on ferroptosis signature genes. (**F**) Violin plot comparing the proliferation signature scores between Cluster 1 and Cluster 2. (**G**) Violin plot comparing the stemness scores between Cluster 1 and Cluster 2. (**H**) t-SNE plot assessing the stemness features of malignant cells based on CytoTRACE. (**I**) t-SNE plot defining the ferroptosis-related malignant (FM) cell group and non-ferroptosis-related malignant (NFM) cell group. (**J**) Violin plot comparing the expression levels of the core gene TCF4 between the FM and NFM groups. (**K**) Gene clustering dendrogram and module color distribution constructed by high-dimensional weighted gene co-expression network analysis (hdWGCNA). (**L**) Heatmap showing the correlation between hdWGCNA modules and the FM/NFM phenotypes. (**M**) Scatter plot showing the high positive correlation between module membership of genes in the turquoise module and gene significance for the FM phenotype. The red dashed lines indicate the thresholds used for screening hub genes: the vertical line represents a module membership (MM) of 0.80, and the horizontal line represents a gene significance (GS) of 0.50. Genes in the upper-right quadrant (MM ≥ 0.80 and GS ≥ 0.50) were considered hub genes that are both highly connected within the turquoise module and strongly associated with the FM phenotype. (**N**) Bubble plot of KEGG pathway enrichment analysis for genes within the turquoise core module. (**O**) Violin plot comparing N-glycosylation levels between the NFM and FM groups. (**P**) Violin plot comparing O-glycosylation levels between the NFM and FM groups. (**Q**) Network plot evaluating the communication interactions between FM cells and other cell types in the TME based on CellChat. The statistical difference was assessed through two-sided Mann–Whitney U test in (**F**,**G**,**J**,**O**,**P**). ns, *p* > 0.05; *, *p* < 0.05; **, *p* < 0.01; ***, *p* < 0.001; ****, *p* < 0.0001.

**Figure 4:**
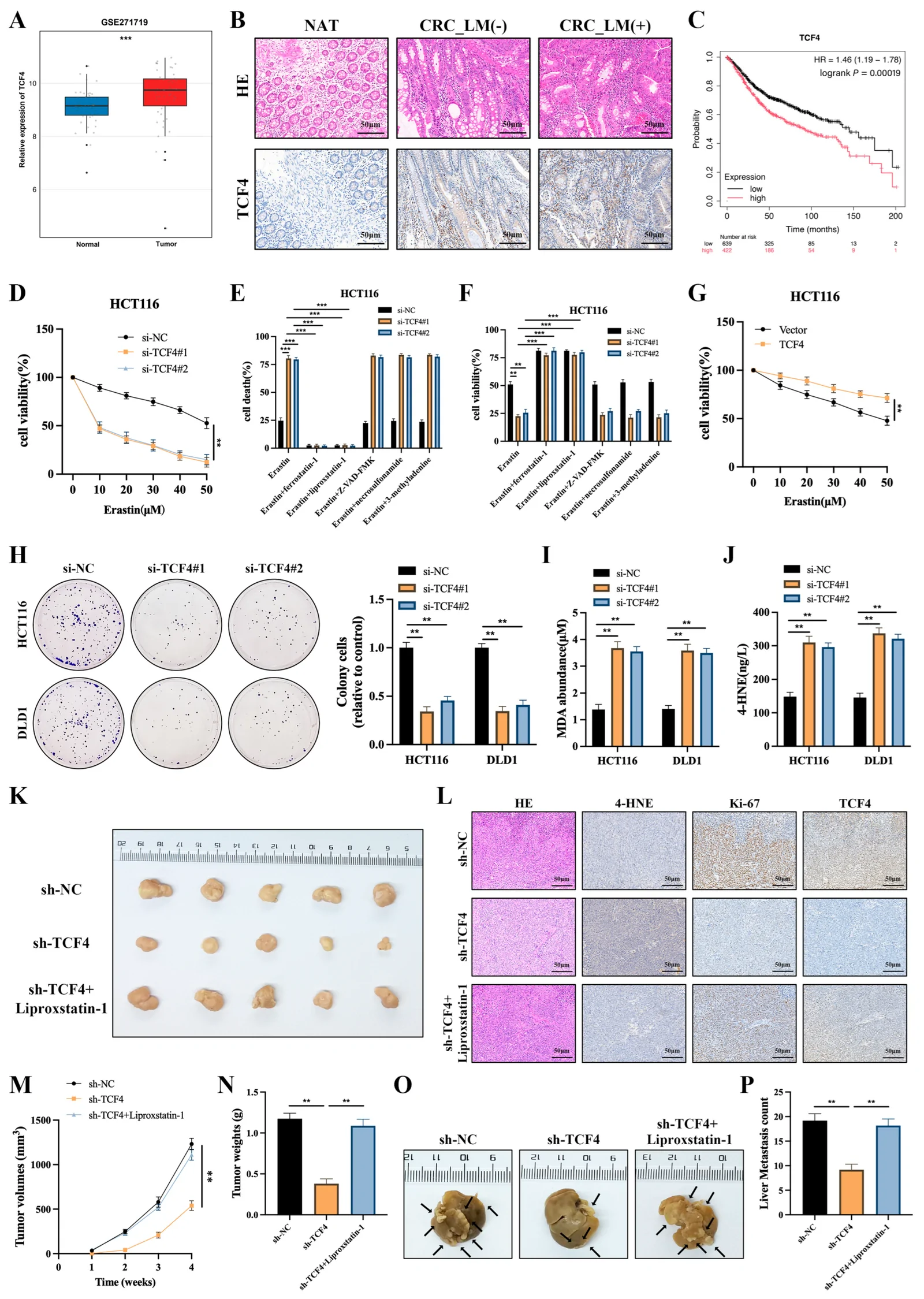
TCF4 promotes ferroptosis resistance and facilitates colorectal cancer liver metastasis. (**A**) TCF4 expression data from the GEO database for colorectal cancer were analysed (*n* = 6). (**B**) Representative IHC images of TCF4 expression in LM-positive or LM-negative CRC tissues and NATs (*n* = 20). Scale bar, 50 μm. (**C**) The overall survival for CRC patients was analyzed using Kaplan–Meier curves (log−rank test). (**D**) Cell viability of HCT116 expressing si−NC or si−TCF4 treated with different concentrations of erastin for 24 h (*n* = 3). (**E**) Cell death measurement of HCT116 expressing si−NC or si−TCF4 treated with erastin (20 μM) in the absence or presence of ferrostatin−1 (2 μM), liproxstatin−1 (1 μM), Z−VAD−FMK (10 μM), necrosulfonamide (0.5 μM), or 3−methyladenine (250 μM) for 24 h (*n* = 3). (**F**) Cell viability measurement of HCT116 expressing si−NC or si−TCF4 treated with erastin (50 μM) in the absence or presence of ferrostatin−1 (2 μM), liproxstatin−1 (1 μM), Z−VAD−FMK (10 μM), necrosulfonamide (0.5 μM), or 3−methyladenine (250 μM) for 24 h (*n* = 3). (**G**) Cell viability of HCT116 expressing Vector or TCF4 treated with different concentrations of erastin for 24 h (*n* = 3). (**H**) Colony formation of CRC cells expressing si−NC or si−TCF4 (*n* = 3). (**I**) MDA production in CRC cells expressing si−NC or si−TCF4 (*n* = 3). (**J**) 4−HNE production in CRC cells expressing si−NC or si−TCF4 (*n* = 3). (**K**) Images of xenograft tumours harvested from nude mice (*n* = 6). (**L**) IHC staining for 4−HNE, Ki67 and TCF4 was measured in xenograft tumours harvested from nude mice (*n* = 6). Scale bar, 50 µm. (**M**) Tumor volume summary for mice, measured every 7 days (*n* = 6). (**N**) Nude mice were examined for tumor weights (*n* = 6). (**O**) Representative livers were acquired from the nude mice (*n* = 6). (**P**) Metastatic nodules in mouse livers were examined (*n* = 6). The statistical difference was assessed through two-sided Mann−Whitney U test in (**A**); one-way ANOVA followed by Dunnett tests in (**D**–**F**,**H**–**J**,**M**,**N**,**P**); and an unpaired Student’s *t*-test in (**G**). Error bars show the SD from three independent experiments. **, *p* < 0.01; ***, *p* < 0.001.

**Figure 5:**
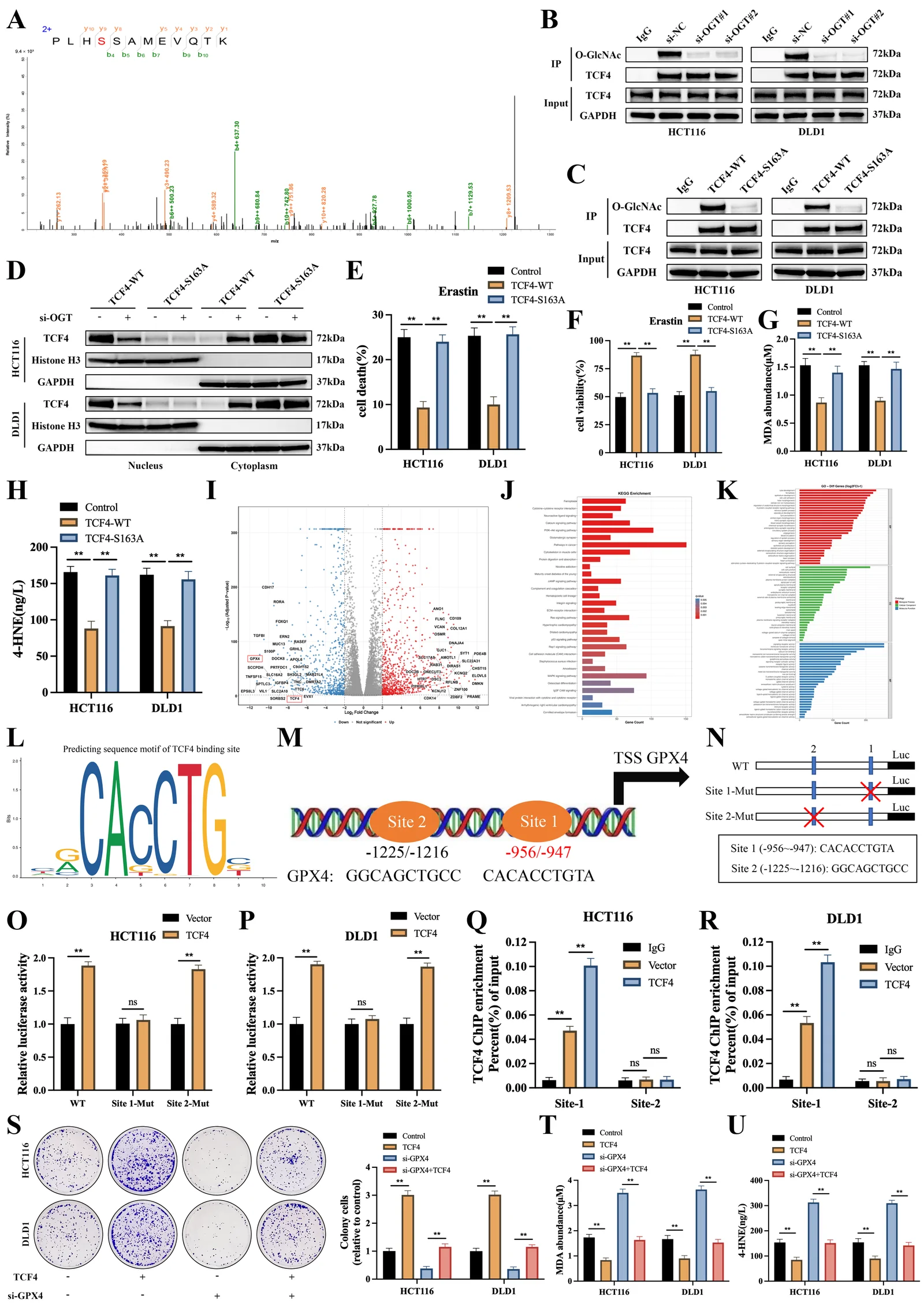
TCF4 regulates ferroptosis via O−GlcNAcylation at serine 163 and transcriptional control of GPX4 in colorectal cancer cells. (**A**) Detection of the O−GlcNAcylation site on TCF4. TCF4 was purified from HCT116 cells and analyzed by LC−MS/MS. The sole O-GlcNAcylation site of TCF4 was identified at Ser163. The score of Mass spectrometry is 102. (**B**) Cell lysates of CRC cells were immunoprecipitated with anti-TCF4 or control IgG, followed by immunoblotting (*n* = 3). (**C**) Immunoblotting of O−GlcNAcylation levels of TCF4−WT and TCF4−S163A after immunoprecipitation with anti−TCF4 (*n* = 3). (**D**) Western blot analyses were performed to examine the nuclear and cytoplasmic localization of TCF4 in CRC cells under different treatment conditions (*n* = 3). (**E**–**H**) Ferroptotic events were evaluated in CRC cells transfected with the indicated plasmids: cell death (**E**), cell viability (**F**), MDA levels (**G**), and 4−HNE levels (**H**) (*n* = 3). (**I**) Volcano plots of upregulated and downregulated genes in TCF4 siRNA compared to those in siRNA con. (**J**) KEGG enrichment analysis of the number of pathways at the mRNA level. (**K**) Gene ontology of biological processes at the mRNA level. (**L**) Enriched motifs of TCF4 binding sites predicted using JASPAR. (**M**) Schematic model of TCF4 binding sequences in the GPX4 promoter region predicted using JASPAR and PROMO. (**N**) Schematic plot of the pGL3−Basic vector containing wild−type and two mutated motifs of the GPX4 promoter. (**O**,**P**) Relative activities of the WT and Mut luciferase reporters (*n* = 3). (**Q**,**R**) ChIP−qPCR analysis of TCF4−enriched chromatin in HCT116 and DLD1 cells (*n* = 3). (**S**) Colony formation of CRC cells expressing si−NC or si−GPX4 and transfected with the TCF4 plasmid (*n* = 3). (**T**) MDA production in CRC cells expressing si−NC or si−GPX4 and transfected with the TCF4 plasmid (*n* = 3). (**U**) 4−HNE production in CRC cells expressing si−NC or si−GPX4 and transfected with the TCF4 plasmid (*n* = 3). The statistical difference was assessed through one-way ANOVA followed by Dunnett tests in (**E**–**H**,**Q**,**R**); and unpaired Student’s *t*-test in (**O**,**P**,**S**–**U**). Error bars show the SD from three independent experiments. ns, *p* > 0.05; **, *p* < 0.01. The uncropped blots are shown in Supplementary Materials.

**Figure 6:**
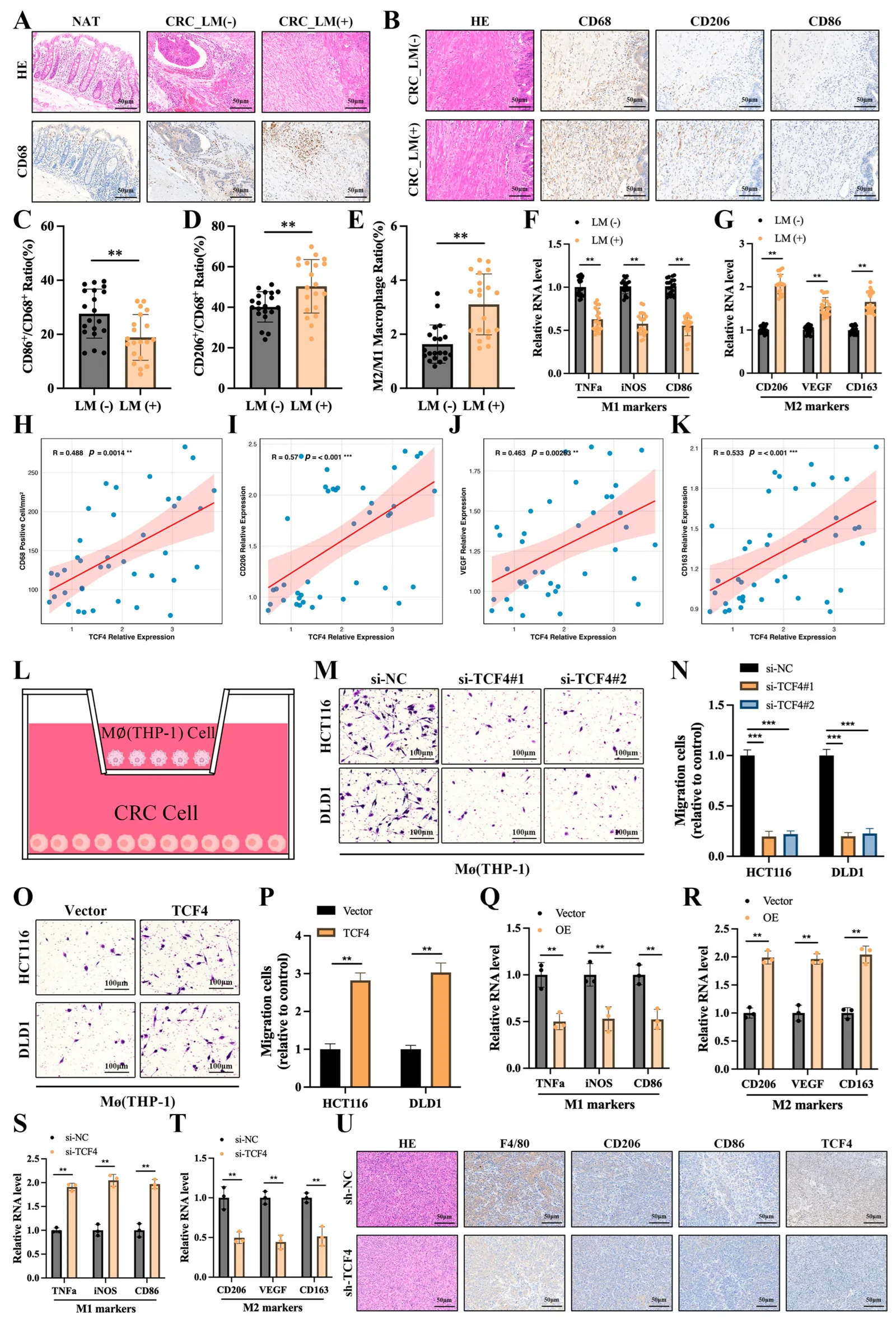
TCF4 promotes macrophage recruitment and M2−like polarization in colorectal cancer. (**A**) Representative IHC staining of CD68 expression in human colorectal cancer tissue collected from NAT, CRC_LM−negative and CRC_LM−positive patients (*n* = 20). Scale bar, 50 μm. (**B**) Representative IHC staining of CD68, CD206 and CD86 expression in human colorectal cancer tissues collected from CRC_LM−negative and CRC_LM-positive patients (*n* = 20). Scale bar, 50 μm. (**C**) Quantification of M1 macrophage (CD86−stained cells)/total TAMs (CD68−stained cells) ratio in human colorectal cancer tissues collected from CRC_LM−negative and CRC_LM−positive patients (*n* = 20). (**D**) Quantification of the M2 macrophage (CD206−stained cells)/total TAMs (CD68−stained cells) ratio in human colorectal cancer tissues collected from CRC_LM−negative and CRC_LM−positive patients (*n* = 20). (**E**) M2/M1 macrophage ratio in human samples (*n* = 20). (**F**) Relative mRNA expression of TNFa, iNOS, CD86 in human colorectal cancer samples (*n* = 20). (**G**) Quantitative PCR results of CD206, VEGF, and CD163 expression in human colorectal cancer samples (*n* = 20). (**H**) Spearman correlation coefficient and *p*-value between TCF4 mRNA expression and CD68-positive cell density in colorectal cancer samples. (**I**–**K**) Spearman correlation coefficient and *p*-value between TCF4 mRNA expression and CD206, VEGF, CD163 mRNA expression. (**L**) Schematic diagram of the THP-1 cells and CRC cells coculture system. (**M**–**P**) Transwell migration assay of THP-1 cells cocultured with indicated CRC cells (*n* = 3). Scale bar, 100 μm. (**Q**) Relative mRNA expression of TNFa, iNOS, CD86 in THP-1 cells (*n* = 3). (**R**) Relative mRNA expression of CD206, VEGF, CD163 in THP-1 cells (*n* = 3). (**S**) Relative mRNA expression of TNFa, iNOS, CD86 in THP-1 cells (*n* = 3). (**T**) Relative mRNA expression of CD206, VEGF, CD163 in THP-1 cells (*n* = 3). (**U**) IHC staining for CD68, CD206, CD86 and TCF4 was measured in xenograft tumours harvested from nude mice (*n* = 6). Scale bar, 50 µm. The statistical difference was assessed through two-sided Mann–Whitney U test in (**C**–**G**); one-way ANOVA followed by Dunnett tests in (**N**); and an unpaired two-sided Student’s *t*-test in (**P**–**T**). Error bars show the SD from three independent experiments. **, *p* < 0.01; ***, *p* < 0.001.

**Figure 7:**
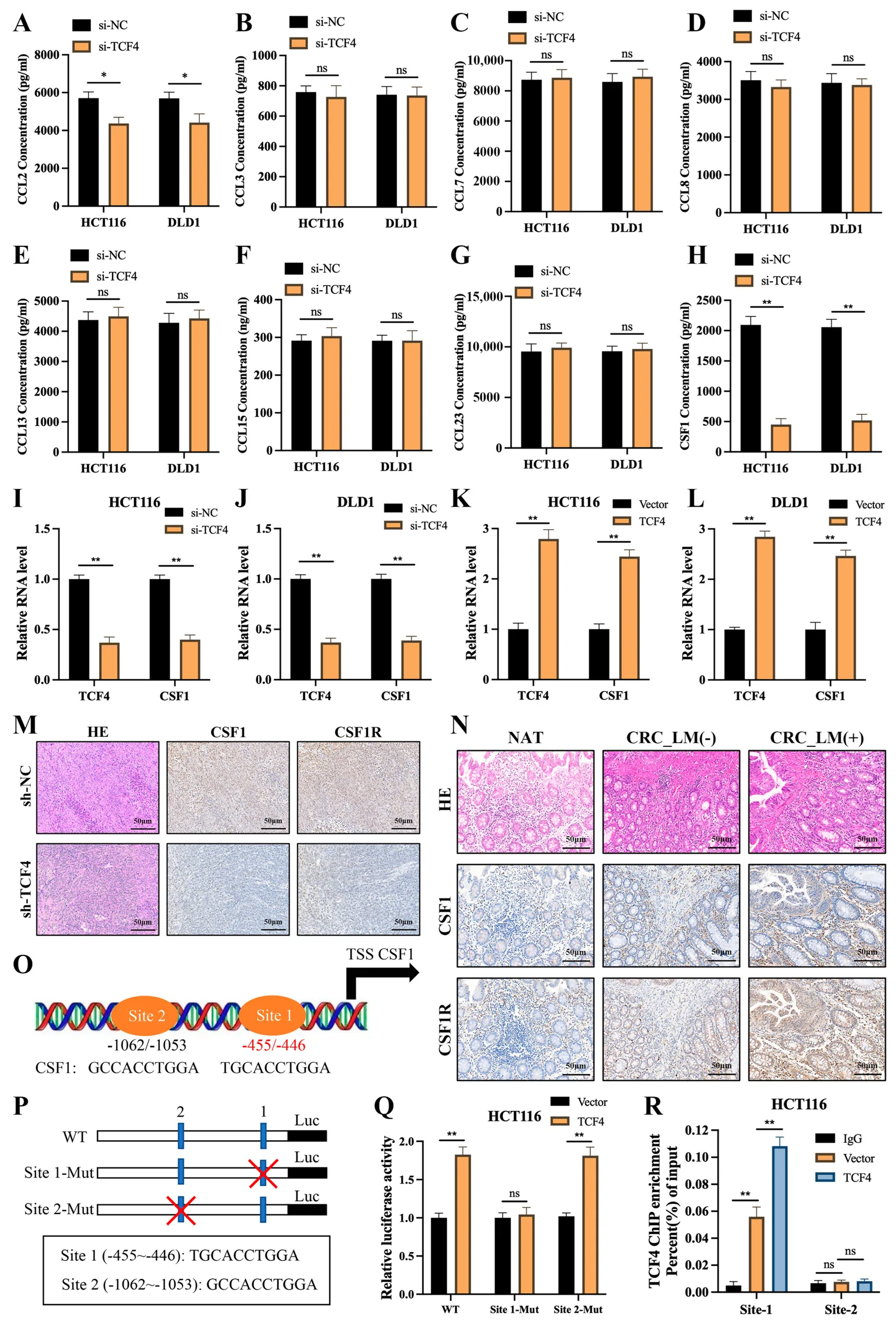
TCF4 induces TAM recruitment and polarization through regulation of the CSF1/CSF1R axis. (**A**–**H**) Concentrations of CCL2, CCL3, CCL7, CCL8, CCL13, CCL15, CCL23, and CSF1 in CRC cells expressing si−NC or si−TCF4 (*n* = 3). (**I**–**L**) Relative TCF4 and CSF1 expressions were detected in HCT116 and DLD1 cells using qRT−PCR (*n* = 3). (**M**) IHC staining for CSF1 and CSF1R was measured in xenograft tumours harvested from nude mice (*n* = 6). Scale bar, 50 µm. (**N**) Representative IHC staining of CSF1 and CSF1R expression in human colorectal cancer tissue collected from NAT, CRC_LM−negative and CRC_LM−positive patients (*n* = 20). Scale bar, 50 μm. (**O**) Schematic model of TCF4 binding sequences in the CSF1 promoter region predicted using JASPAR and PROMO. (**P**) Schematic plot of the pGL3-Basic vector containing wild-type and two mutated motifs of the CSF1 promoter. (**Q**) Relative activities of the WT and Mut luciferase reporters (*n* = 3). (**R**) ChIP-qPCR analysis of TCF4−enriched chromatin in HCT116 cells (*n* = 3). The statistical difference was assessed through unpaired two-sided Student’s *t*-test in (**A**–**L**,**Q**); and one-way ANOVA followed by Dunnett tests in (**R**). Error bars show the SD from three independent experiments. ns, *p* > 0.05; *, *p* < 0.05; **, *p* < 0.01.

## Data Availability

The datasets used or analyzed during the current study are available from the corresponding author on reasonable request.

## Supplementary Materials

The following supporting information can be downloaded at: https://www.mdpi.com/article/10.3390/cancers18193105/s1, File S1: Supplemental Methods and Materials; Figure S1. Identification of ferroptosis-related genes (FRGs) and molecular subclusters in colorectal cancer based on the TCGA-COAD cohort. Figure S2. Construction and validation of a prognostic risk model based on ferroptosis-related genes in colorectal cancer; Figure S3. Identification of liver metastasis (LM)-associated genes and construction of a diagnostic nomogram for LM in colorectal cancer.; Figure S4. Identification of ferroptosis-associated malignant cell subclusters and characterization of their metabolic and glycosylation features through single-cell and pseudotime trajectory analyses; Figure S5. TCF4 regulates ferroptosis sensitivity in colorectal cancer cells; Figure S6. OGT-mediated O-GlcNAcylation of TCF4 at Ser163 regulates ferroptosis in colorectal cancer cells; Figure S7. O-GlcNAcylation and TCF4 regulate GPX4 expression and ferroptosis sensitivity in colorectal cancer cells; Figure S8. Correlation between TCF4 expression and immune cell infiltration in colorectal cancer; Figure S9. O-GlcNAcylation of TCF4 at Ser163 in CRC cells promotes macrophage M2 polarization in co-culture systems; Figure S10. O-GlcNAcylation of TCF4 at Ser163 promotes CSF1 expression and macrophage M2 polarization in colorectal cancer cells; Table S1. Primers and siRNAs used in the experiments; Table S2. Antibodies used in the experiments.

## Abbreviations

TCF4	transcription factor 4
GPX4	glutathione peroxidase 4
CSF1	colony-stimulating factor 1
4-HNE	4-hydroxynonenal
AUC	area under the curve
CAMs	cell adhesion molecules
ChIP	chromatin immunoprecipitation
CRC	colorectal cancer
CRLM	colorectal cancer liver metastasis
ECM	extracellular matrix
ELISA	enzyme-linked immunosorbent assay
FDR	false discovery rate
FRGs	ferroptosis-related genes
GEO	Gene Expression Omnibus
GO	Gene Ontology
GSEA	gene set enrichment analysis
hdWGCNA	high-dimensional weighted gene co-expression network analysis
IHC	immunohistochemistry
KEGG	Kyoto Encyclopedia of Genes and Genomes
LASSO	least absolute shrinkage and selection operator
MDA	malondialdehyde
MDSCs	myeloid-derived suppressor cells
mRNA	messenger RNA
NAT	normal adjacent tissue
NMF	non-negative matrix factorization
O-GlcNAc	O-linked β-N-acetylglucosamine
OS	overall survival
PCA	principal component analysis
PCR	polymerase chain reaction
PTM	post-translational modification
qRT–PCR	quantitative reverse transcription polymerase chain reaction
RF	random forest
RNA-seq	RNA sequencing
scRNA-seq	single-cell RNA sequencing
siRNA	small interfering RNA
TAMs	tumor-associated macrophages
TCGA-COAD	The Cancer Genome Atlas–Colon Adenocarcinoma
t-SNE	t-distributed stochastic neighbor embedding
TME	tumor microenvironment
UMAP	uniform manifold approximation and projection
