# Supplementary material for: O-GlcNAcylated TCF4 Drives Ferroptosis Resistance and Tumor-Associated Macrophage Infiltration to Promote Colorectal Cancer Liver Metastasis
Source: Cancers (Basel). 2026 Sep 24;18(19):3105. doi: 10.3390/cancers18193105 (PMC13664046; doi:10.3390/cancers18193105)

Original WB images

Original images 1. Figure 5B anti-TCF4 (IP) (HCT116)

IB: anti-O-GlcNAc (IgG vs siNC vs siOGT1/2)

kDa
180
130
100
72
55
43
34
26
15

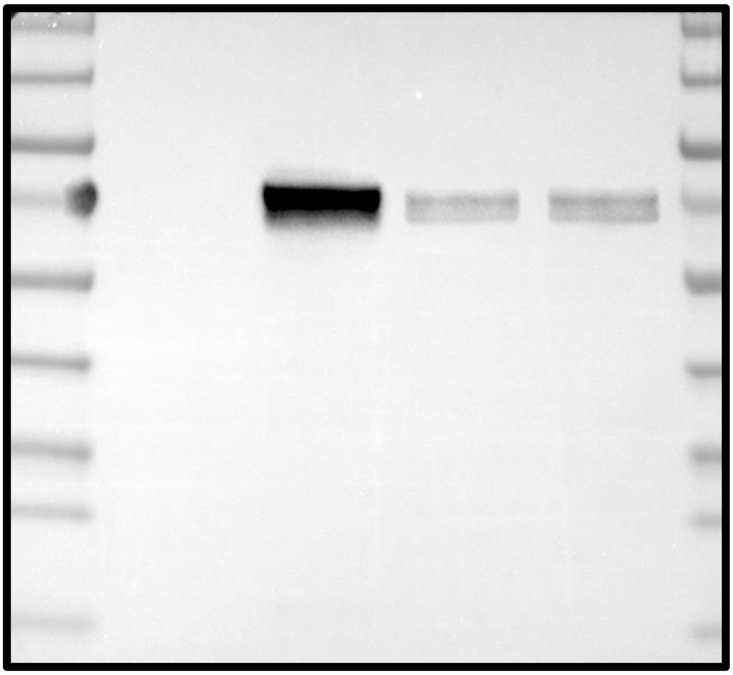

1: 100: 19: 23

Original images 2. Figure 5B anti-TCF4 (IP) (HCT116)

IB: anti-TCF4 (IgG vs siNC vs siOGT1/2)

kDa
180
130
100
72
55
43
34
26
15

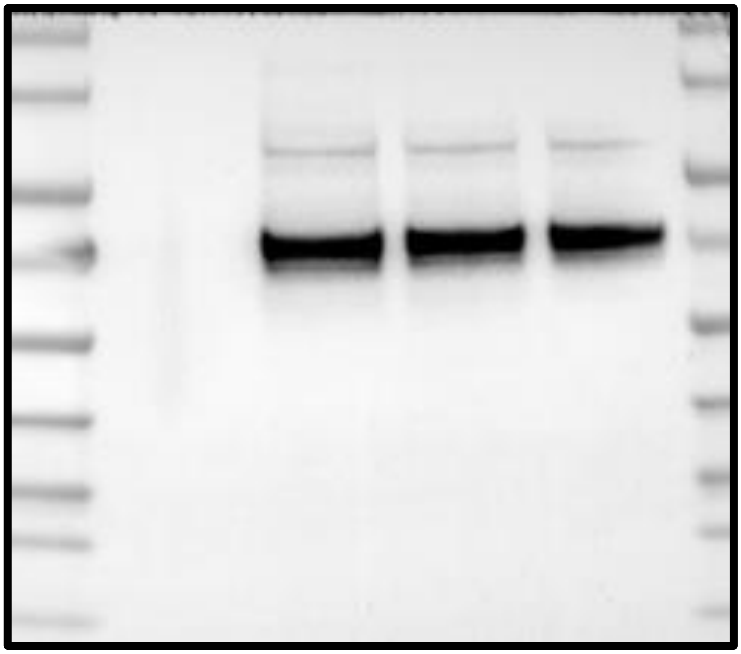

4: 100: 101: 103

Original images 3. Figure 5B anti-TCF4 (Input) (HCT116)

IB: anti-TCF4 (IgG vs siNC vs siOGT1/2)

kDa
100
72
55

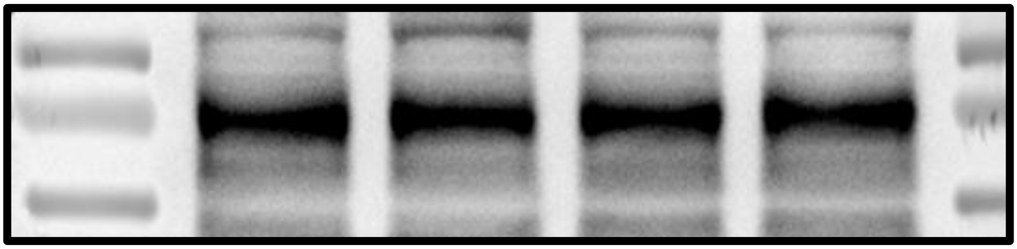

100: 101: 97: 92

Original images 4. Figure 5B anti-GAPDH (Input) (HCT116)

IB: anti-GAPDH (IgG vs siNC vs siOGT1/2)

kDa
43
34
26

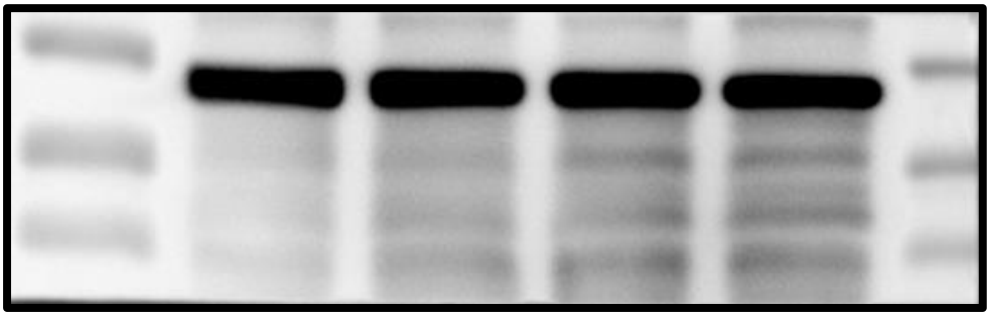

100: 104: 102: 98

Original images 5. Figure 5B anti-TCF4 (IP) (DLD1)

IB: anti-O-GlcNAc (IgG vs siNC vs siOGT1/2)

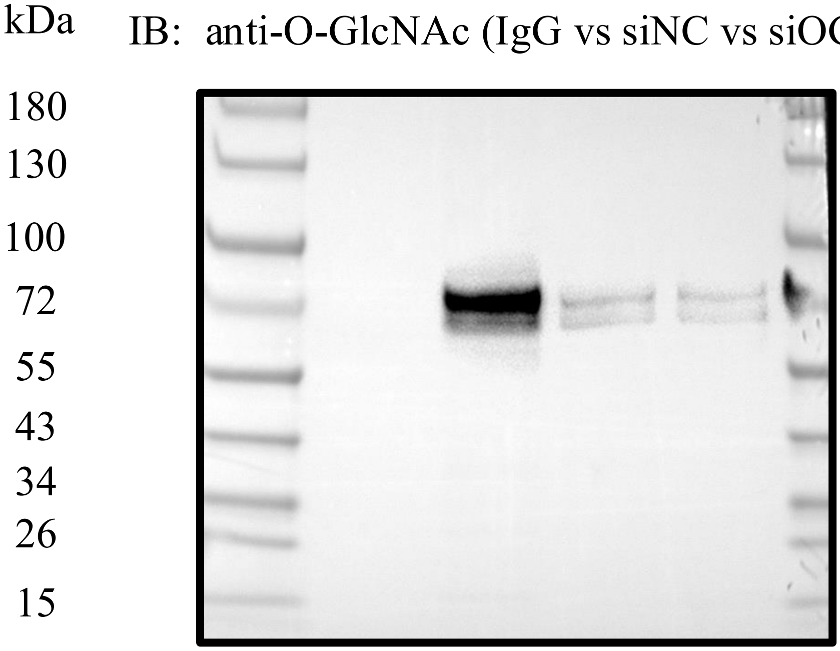

1: 100: 17: 13

Original images 6. Figure 5B anti-TCF4 (IP) (DLD1)

IB: anti-TCF4 (IgG vs siNC vs siOGT1/2)

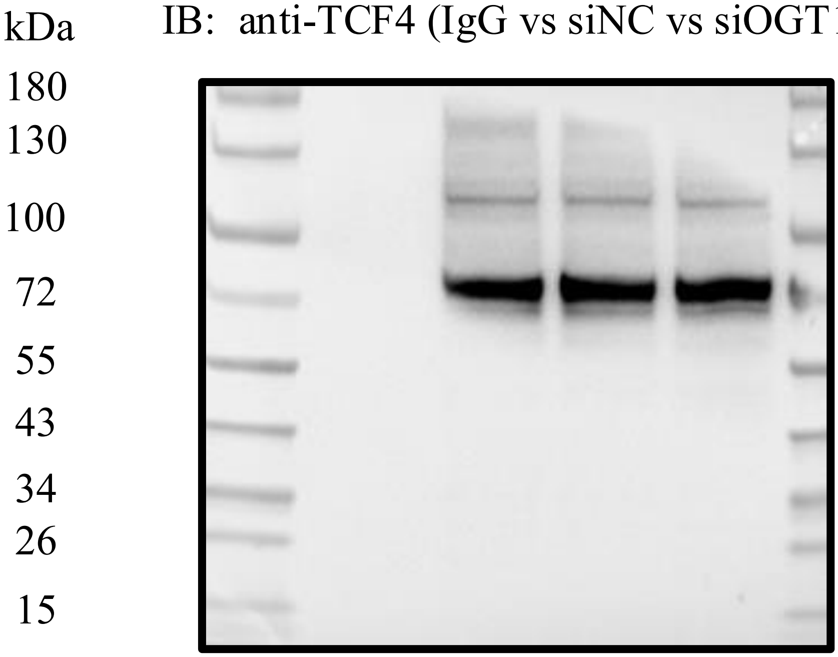

2: 100: 103: 104

Original images 7. Figure 5B anti-TCF4 (Input) (DLD1)

IB: anti-TCF4 (IgG vs siNC vs siOGT1/2)

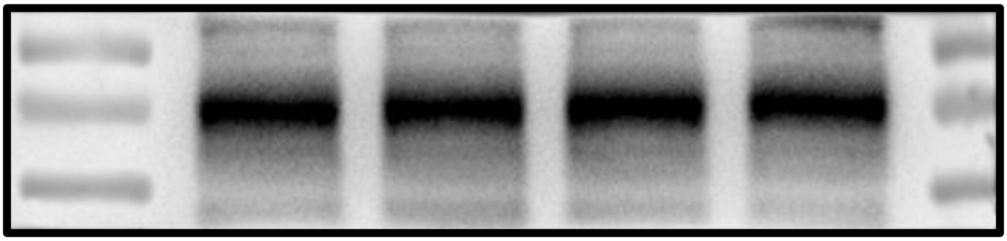

100: 104: 106: 99

Original images 8. Figure 5B anti-GAPDH (Input) (DLD1)

IB: anti-GAPDH (IgG vs siNC vs siOGT1/2)

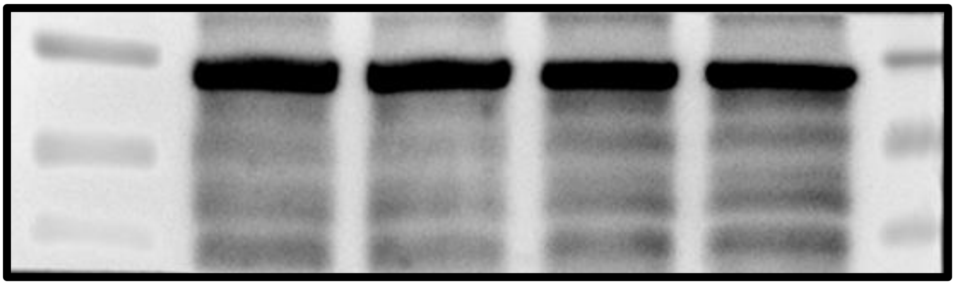

100: 98: 97: 96

Original images 9. Figure 5C anti-TCF4 (IP) (HCT116)

IB: anti-O-GlcNAc (IgG vs TCF4-WT vs TCF4-S163A)

kDa
180
130
100
72
55
43
34
26
15

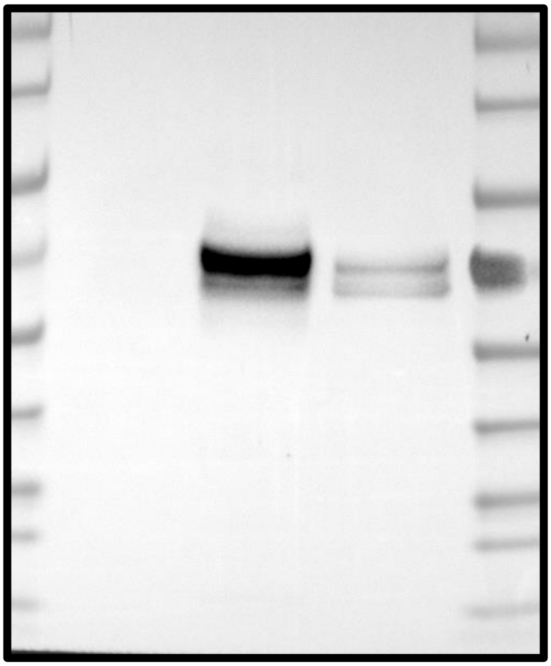

2: 100: 15

Original images 10. Figure 5C anti-TCF4 (IP) (HCT116)

IB: anti-TCF4 (IgG vs TCF4-WT vs TCF4-S163A)

kDa
180
130
100
72
55
43
34
26
15

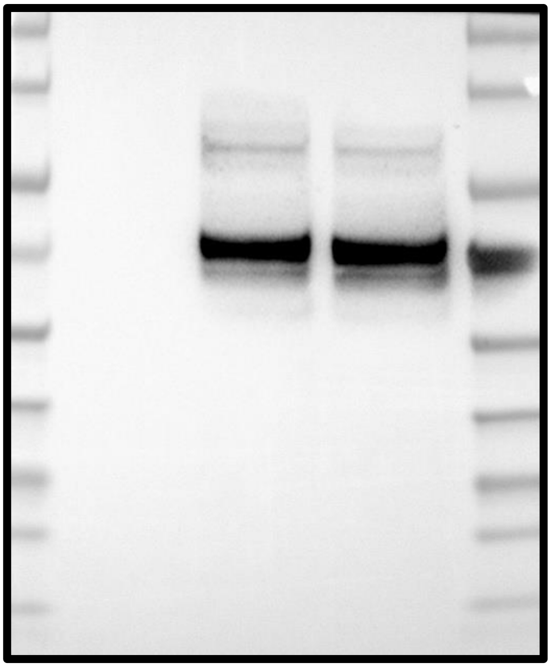

1: 100: 103

Original images 11. Figure 5C anti-TCF4 (Input) (HCT116)

IB: anti-TCF4 (IgG vs TCF4-WT vs TCF4-S163A)

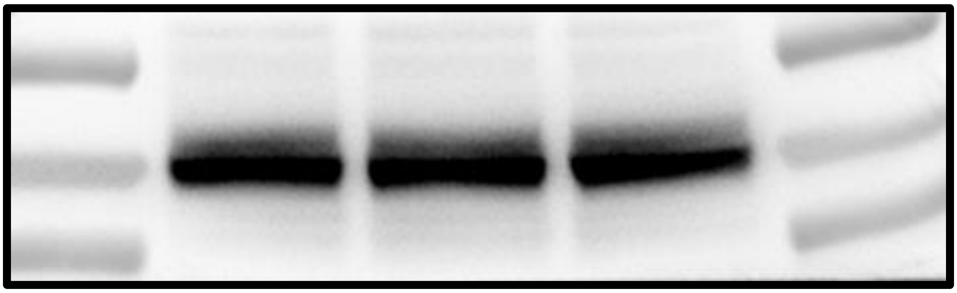

100: 105: 102

Original images 12. Figure 5C anti-GAPDH (Input) (HCT116)

IB: anti-GAPDH (IgG vs TCF4-WT vs TCF4-S163A)

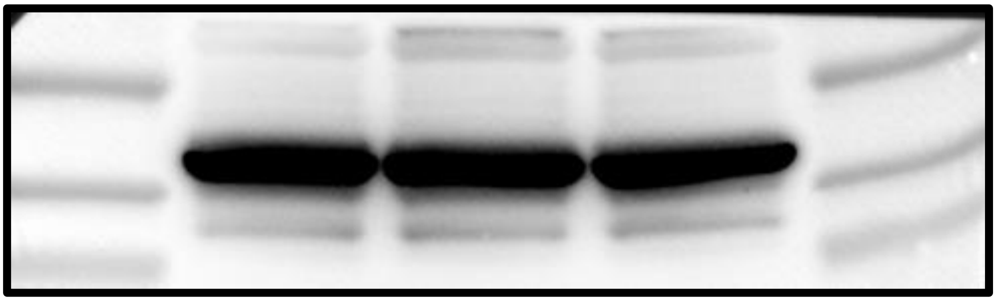

100: 101: 99

Original images 13. Figure 5C anti-TCF4 (IP) (DLD1)

IB: anti-O-GlcNAc (IgG vs TCF4-WT vs TCF4-S163A)

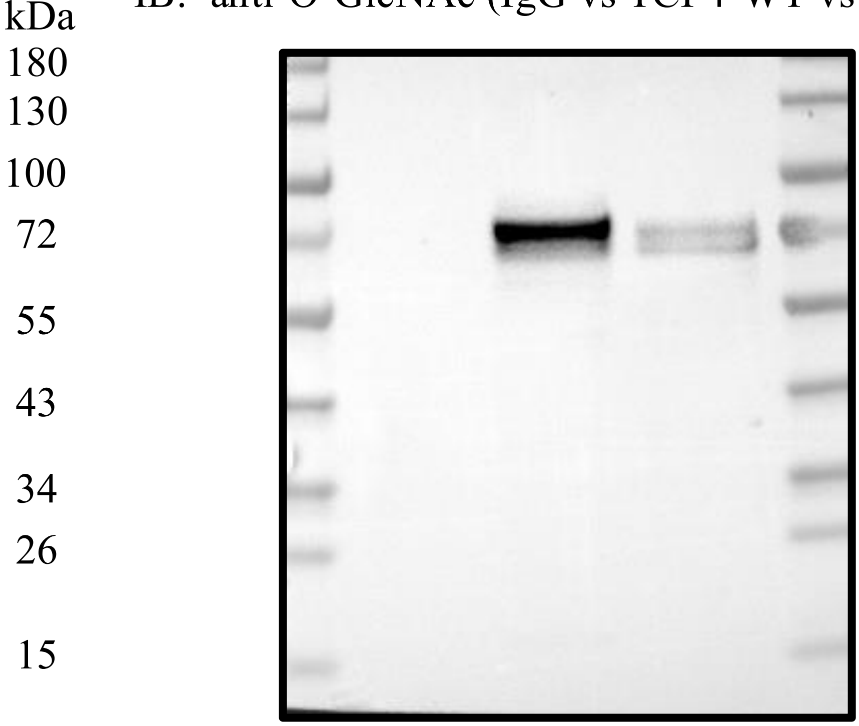

1: 100: 14

Original images 14. Figure 5C anti-TCF4 (IP) (DLD1)

IB: anti-TCF4 (IgG vs TCF4-WT vs TCF4-S163A)

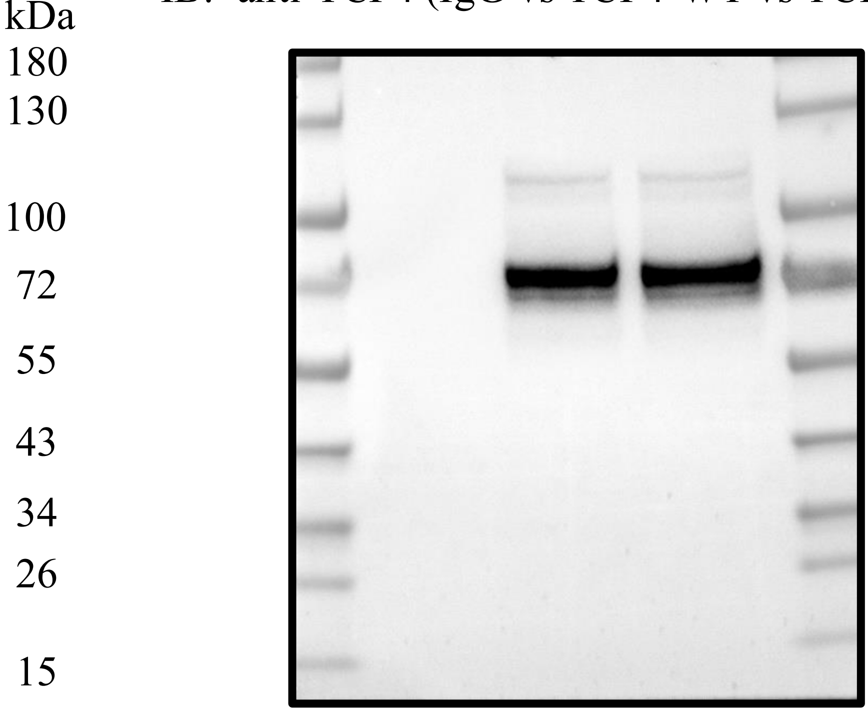

2: 100: 97

Original images 15. Figure 5C anti-TCF4 (Input) (DLD1)

IB: anti-TCF4 (IgG vs TCF4-WT vs TCF4-S163A)

kDa

100

72

55

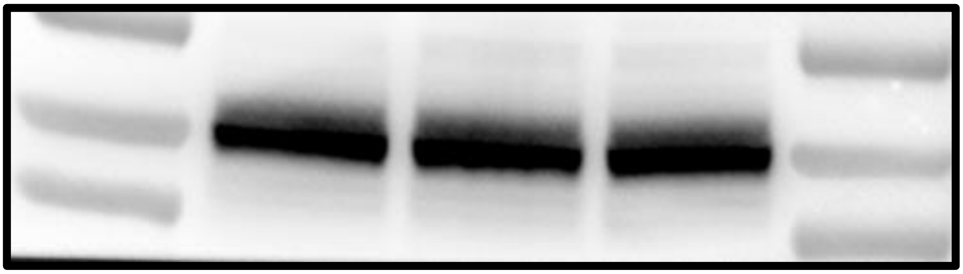

100: 95: 104

Original images 16. Figure 5C anti-GAPDH (Input) (DLD1)

IB: anti-GAPDH (IgG vs TCF4-WT vs TCF4-S163A)

kDa

43

34

26

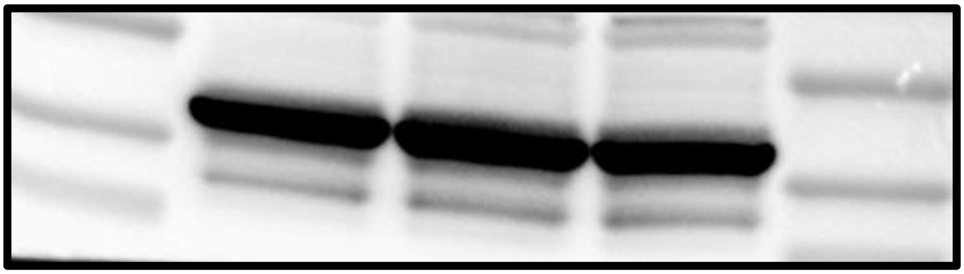

100: 108: 103

Original images 17. Figure 5D anti-TCF4 (HCT116)

IB: anti-TCF4 (Nucleus vs Cytoplasm, TCF4-WT vs TCF4-S163A)

kDa
100
72
55

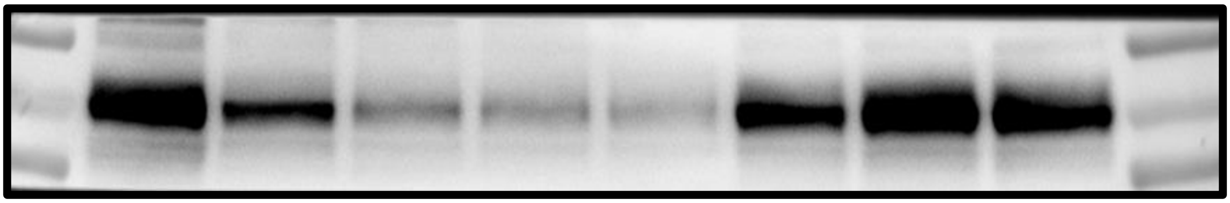

100: 35: 17: 15: 12: 74: 103: 96

Original images 18. Figure 5D anti-Histone H3 (HCT116)

kDa IB: anti-Histone H3 (Nucleus vs Cytoplasm, TCF4-WT vs TCF4-S163A)

26
15
10

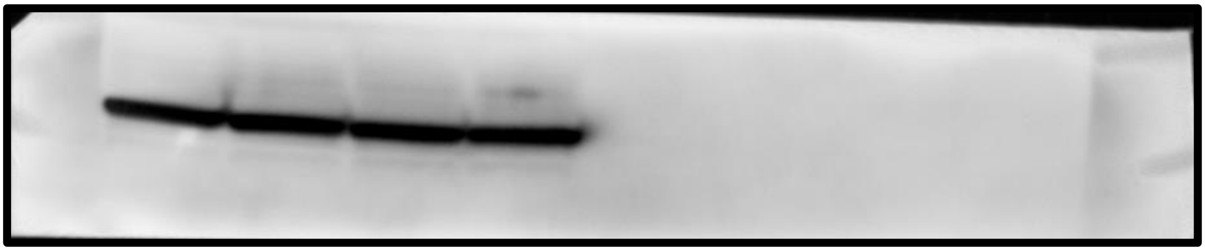

100: 104: 102: 98: 1: 3: 3: 2

Original images 19. Figure 5D anti-GAPDH (HCT116)

IB: anti-GAPDH (Nucleus vs Cytoplasm, TCF4-WT vs TCF4-S163A)

kDa
55
43
34

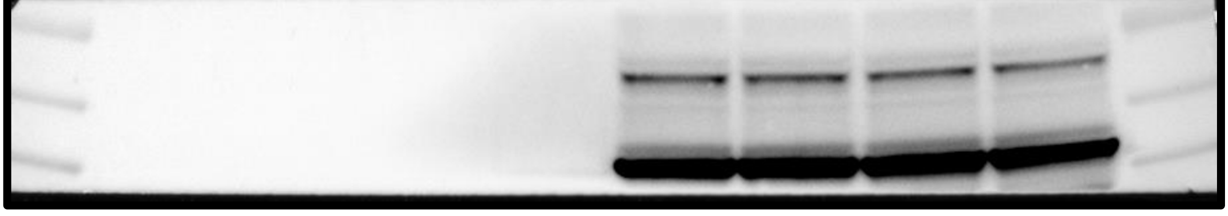

1: 1: 2: 4: 100: 104: 106: 97

Original images 20. Figure 5D anti-TCF4 (DLD1)

IB: anti-TCF4 (Nucleus vs Cytoplasm, TCF4-WT vs TCF4-S163A)

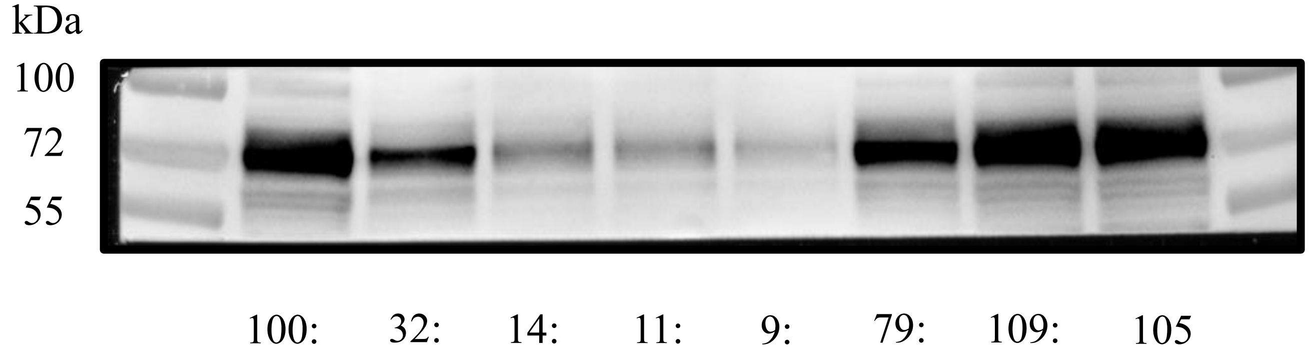

Original images 21. Figure 5D anti-Histone H3 (DLD1)

kDa IB: anti-Histone H3 (Nucleus vs Cytoplasm, TCF4-WT vs TCF4-S163A)

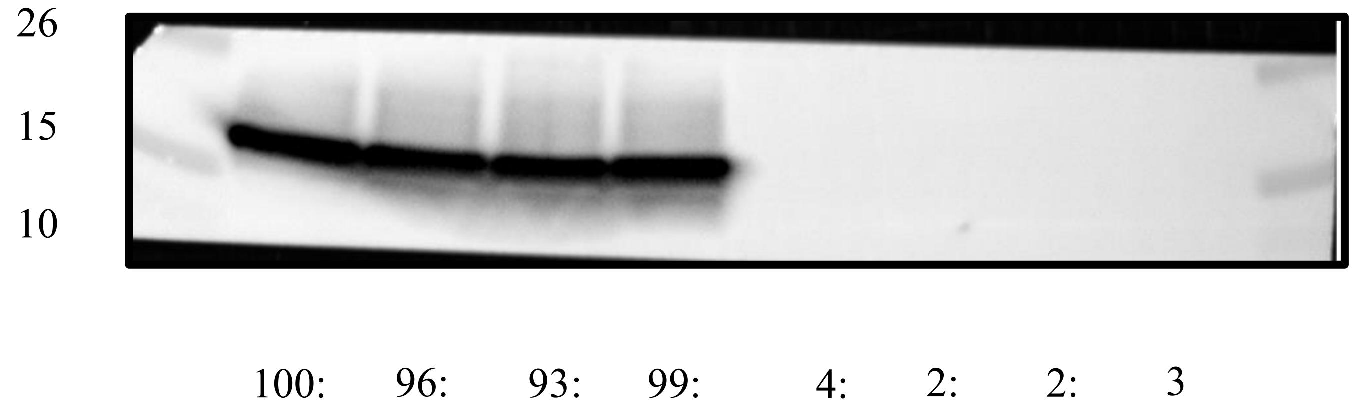

Original images 22. Figure 5D anti-GAPDH (DLD1)

IB: anti-GAPDH (Nucleus vs Cytoplasm, TCF4-WT vs TCF4-S163A)

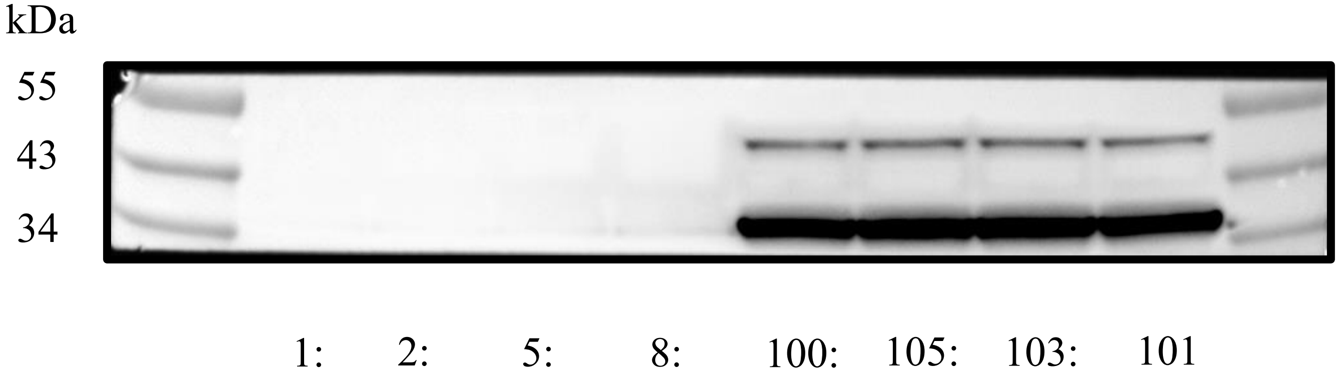

Supplement: Supplementary file 1 [file cancers-18-03105-s001.zip › cancers-4554184-original-images.pdf]
