# Supplementary material for: O-GlcNAcylated TCF4 Drives Ferroptosis Resistance and Tumor-Associated Macrophage Infiltration to Promote Colorectal Cancer Liver Metastasis
Source: Cancers (Basel). 2026 Sep 24;18(19):3105. doi: 10.3390/cancers18193105 (PMC13664046; doi:10.3390/cancers18193105)
Supplement: Supplementary file 1 [file cancers-18-03105-s001.zip › Supplemental Methods and Materials.pdf]

Supplemental Methods and Materials

## 1. Data Acquisition and Processing

This study downloaded the transcriptome data and matched clinical data of the colon adenocarcinoma (COAD) cohort from The Cancer Genome Atlas (TCGA) database (https://portal.gdc.cancer.gov/) and strictly excluded samples with a survival time of $\leqslant$ 0 days or missing survival information. To perform external validation and multi-dimensionally explore the transcriptional features of colorectal cancer (CRC), multiple datasets were included from the Gene Expression Omnibus (GEO) database (www.ncbi.nlm.nih.gov/geo/): 566 tumor samples and their clinical data from GSE39582 were selected as the external validation set; sequencing data of 49 paired tumor and normal tissues from GSE271719 were included; to investigate factors related to CRC liver metastasis, data of 190 primary CRC and 55 liver metastasis samples were obtained from GSE68468; meanwhile, to deeply decipher the microenvironment evolution during the liver metastasis process, colorectal cancer liver metastasis samples from the single-cell sequencing dataset GSE164522 were included. In addition, with a threshold of Relevance score > 1, 1522 ferroptosis-related genes were screened from the GeneCards database (https://www.genecards.org/). Differential expression analysis was performed using the R package "DESeq2", with the significance threshold set at $|\log2FC| > 1$ and $FDR < 0.05$.

## 2. Non-negative Matrix Factorization (NMF) Clustering Analysis

Unsupervised clustering subtyping of the transcriptome data from the TCGA-COAD cohort was performed using the non-negative matrix factorization algorithm in the R package "NMF". By comprehensively evaluating the attenuation trends of metrics such as Cophenetic correlation and Dispersion, the optimal number of clusters was determined to be $k = 2$. Subsequently, principal component analysis (PCA), t-distributed stochastic neighbor embedding (t-SNE), and uniform manifold approximation and projection (UMAP) dimensionality reduction analyses were conducted utilizing the R packages "broom", "Rtsne", and "umap", respectively, to intuitively visualize and verify the reliability and distinguishability of the clustering

results. Furthermore, survival analysis was performed and Kaplan–Meier (K-M) survival curves were plotted using the R packages "survival" and "survminer". Prognostic differences between groups were evaluated via the Log-rank test, with $p < 0.05$ considered statistically significant.

## 3. Functional Enrichment and Tumor Microenvironment Immune Profile Analysis

The enrichment status of KEGG and Reactome pathways among different subgroups was evaluated using the "GSVA" and "msigdbr" packages. Differentially expressed genes were extracted using "limma", and GO and KEGG enrichment analyses were performed via the "clusterProfiler" package (adjusted $p < 0.05$). In the tumor microenvironment (TME) evaluation, the infiltration proportions of immune cells were quantified by comprehensively utilizing the ssGSEA and CIBERSORT algorithms, and differences between groups were analyzed via the Wilcoxon rank-sum test. Spearman correlation analysis was employed to assess the association between core genes and immune infiltration, and heatmaps were plotted. Additionally, the "estimate" package was used to calculate three TME scores: ImmuneScore, StromalScore, and ESTIMATEScore. After evaluating the differences between groups via the Wilcoxon test, the results were visualized using the " ggpubr" and "ggplot2" packages.

## 4. Weighted Gene Co-expression Network Analysis (WGCNA)

A co-expression network was constructed using the "WGCNA" package to identify relevant trait-associated genes. After removing outlier samples, the network was built based on differentially expressed genes. The scale-free topology fit index ($R^2$) was set to 0.85 to determine the optimal soft-thresholding power ($\beta = 12$). The topological overlap matrix (TOM) was calculated, and average linkage hierarchical clustering was adopted to construct a dendrogram. The dynamic tree cut algorithm was applied to divide modules, with the minimum number of genes per module set to 50 and the threshold for merging similar modules set to 0.2. Finally, by evaluating the Pearson correlation between module eigengenes and the C2 sub-cluster, combined with module membership (MM) and gene significance (GS), key genes within the core modules were screened for downstream analysis.

## 5. Construction of the Prognostic Risk Model

Based on the module feature genes screened through WGCNA, this study constructed a prognostic risk scoring model for colorectal cancer (CRC). First, the study cohort was randomly divided into an internal training set and an internal validation set at a ratio of 7:3, and the GSE39582 dataset (n = 566) was included as an independent external validation set. In the internal training set, the least absolute shrinkage and selection operator (LASSO) Cox regression analysis was performed using the R package "glmnet" to eliminate collinearity among features and prevent model overfitting. Through 10-fold cross-validation, the optimal penalty parameter (λ) was determined based on the minimum partial likelihood deviance, and the optimal feature factors were screened. Subsequently, these factors were incorporated into a multivariate Cox proportional hazards regression analysis to determine the final prognostic model. The risk score for each patient was calculated according to the following formula:

$$RiskScore_i = \sum_j \beta_j \cdot Expr_{\{ij\}}$$

In this formula, $\beta_j$ represents the regression coefficient assigned to gene j, and $Expr_{\{ij\}}$ represents the expression level of gene j in sample i. Based on the median value of the Risk Score in the internal training set, all cohort samples were uniformly stratified into a high-risk group and a low-risk group. Kaplan–Meier survival analysis was performed using the "survival" package to compare the overall survival (OS) between the two groups. Time-dependent ROC curves were utilized to evaluate the accuracy (AUC) of the model in predicting 1-, 3-, and 5-year OS. Furthermore, Cox regression analysis was conducted in combination with variables such as age and clinical stage to verify whether the risk score is an independent prognostic factor. Finally, a comprehensive prognostic nomogram was constructed using the "rms" package, and calibration curves along with cumulative hazard evaluation plots were generated to validate the clinical predictive utility of the model.

## 6. Development of a Diagnostic Model for Colorectal Cancer Liver Metastasis

To explore the underlying mechanisms and diagnostic markers of CRC liver metastasis, this study included the sequencing data of 190 primary CRC and 55 liver metastasis

patients from the GSE68468 dataset. First, differential expression analysis was performed with the threshold set at $|log2FC| > 0.5$ and an adjusted $p < 0.05$ to identify differentially expressed genes associated with liver metastasis. Prior to machine learning analysis, the dataset was normalized. To ensure reproducibility, the dataset was randomly split into training and testing sets at a 7:3 ratio using a fixed random seed (set. Seed (123)). During feature selection utilizing Support Vector Machine (SVM) and Random Forest (RF) algorithms, a 10-fold cross-validation framework was rigorously employed within the training set to prevent overfitting and select the optimal features. Subsequently, two machine learning algorithms, Support Vector Machine (SVM) and Random Forest (RF), were introduced to predict the liver metastasis status of patients based on the differentially expressed genes. By comparing the model residual values and the performance of receiver operating characteristic (ROC) curves, the algorithm with the optimal predictive efficacy was evaluated and selected. Based on the RF algorithm, which demonstrated superior performance, in-depth mining was conducted to screen out the core feature genes for predicting liver metastasis. Furthermore, utilizing the "rms" and "rmda" packages, an exclusive liver metastasis diagnostic nomogram was constructed based on the aforementioned core genes. By plotting calibration curves and assessing the clinical net benefit intervals, the accuracy and clinical practical value of this diagnostic model were comprehensively evaluated.

**7. Single-Cell Transcriptome Analysis**

Based on the GSE164522 dataset, this study deciphered the microenvironmental features of colorectal cancer liver metastasis (CRLM). Rigorous quality control was performed using the "Seurat" package, retaining high-quality cells (a total of 73,904) with the number of detected genes between 300 and 10,000 and a mitochondrial gene expression proportion of < 20%. After global log-normalization, extraction of highly variable feature genes, and batch effect correction using the Harmony algorithm, dimensionality reduction and visualization were performed via principal component analysis (PCA) and the UMAP algorithm. Combining the "FindClusters" unsupervised clustering with canonical markers, a total of 11 major cell populations were identified, including CD4+ T cells, CD8+ T cells, regulatory T cells (Tregs), B cells, macrophages,

NK cells, plasma cells, malignant tumor cells, mast cells, fibroblasts, and dendritic cells. To deeply investigate the mechanistic role of ferroptosis, malignant tumor cells were reclustered based on the previously identified ferroptosis prognostic signature genes, dividing them into non-ferroptosis-related (NFM) and ferroptosis-related (FM) subpopulations. At the functional evaluation level, the "AddModuleScore" and "CytoTRACE" algorithms were utilized to quantify cell proliferation scores, O-glycosylation modification levels, and tumor stemness features, respectively. Subsequently, high-dimensional weighted gene co-expression network analysis (hdWGCNA) was adopted to identify the core modules highly significantly associated with the FM subpopulation, and the "clusterProfiler" package and GSEA were combined to explore their pathway activation status. Finally, pseudotime developmental trajectories were constructed using "Monocle2" to dynamically reconstruct cell fates, and the "CellChat" tool was utilized to comprehensively evaluate the complex communication and interaction networks between FM cells and other cells within the microenvironment.

**8. Patients and human samples**

In this study, tissue specimens were collected from colorectal cancer (CRC) patients at Guangdong Provincial Hospital of Chinese Medicine. The cohort comprised 66 patients with CRC who did not develop liver metastases and 20 patients with CRC who had liver metastases. Inclusion Criteria: (1) Definitive pathological diagnosis of colorectal cancer. (2) Absence of preoperative radiotherapy or chemotherapy. (3) No history of immune-related disorders. (4) Availability of complete clinical data. During the surgical procedures, tumor tissues were obtained. The tissues were then fixed in formalin, rapidly frozen in liquid nitrogen, and stored at -80°C. Two independent pathologists confirmed the tumor nature of all the collected tissue specimens. Clinical tumor characteristics were gathered from clinical reports. Information on sample-related data, such as gender and age, was obtained through face-to-face interviews with the patients. This study was approved by the Medical Ethics Committee of Guangdong Provincial Hospital of Chinese Medicine (Approval No: ZE-2026-120-01).

**9. Quantitative real-time PCR (qRT-PCR)**

Total RNA was extracted from cells and tissues using TRIzol reagent (Invitrogen). HiScript II Q Select RT SuperMix (Vazyme, Nanjing, China) was employed for cDNA synthesis to analyze messenger RNAs (mRNAs). Subsequently, PCR amplification was performed using ChamQ SYBR qPCR Master Mix (Vazyme). The analysis was carried out on the LightCycler 96 system (Roche, Mannheim, Germany). GAPDH served as an internal control for the detection of mRNAs. The relative expression levels were determined using the $2^{-\Delta Ct}$ or $2^{-\Delta\Delta Ct}$ method. All experiments were repeated three times. The primers used are listed in Supplementary Table 1.

**10. Western blot**

Harvested cells were washed with PBS three times, then lysed with RIPA lysis buffer (Thermo Scientific) supplemented with a cocktail of phosphatase inhibitors and protease inhibitors at 4 °C for 30 min. The cell lysate was spun at 12,000 g for 30 min at 4°C, followed by collecting the supernatant and estimating total protein concentration using the BCA kit (CWBio, Taizhou, China). Subsequently, equal amounts of protein were separated by 10% SDS polyacrylamide gel electrophoresis (SDS-PAGE) and transferred onto a polyvinylidene fluoride (PVDF) membrane, which was blocked with 5% BSA for 1 h at room temperature, incubated at 4 °C overnight with primary antibodies, washed three times with Tris-buffered saline containing 0.1% Tween-20, then incubated with horseradish peroxidase-conjugated secondary antibodies for 1 h at room temperature. The immunoblots were detected by the ECL chemiluminescence kit (Thermo Scientific) and quantified using Image J software (NIH). The antibodies used are listed in Supplementary Table 2.

**11. Lentivirus infection and cell transfection**

T To establish stable TCF4-overexpressing or TCF4-knockdown CRC cell lines, the cells were infected with lentiviral particles encoding TCF4 or short hairpin RNA (shRNA) targeting TCF4, which were generated by Genepharma (Shanghai, China). After infection, the cells were selected with puromycin (Sigma-Aldrich, St. Louis, MO, USA) for 2 weeks to obtain stable transfectants.

For cell transfection, $2 \times 10^5$ cells per well were seeded into the 6-well plate and cultured overnight at 37°C in 5% $CO_2$. The indicated plasmids and small interfering

RNAs (siRNAs) (Genepharma) were transfected into cells in the presence of lipofectamine 3000 (Invitrogen, Carlsbad, CA, USA) according to the protocols from manufacturer. qRT-PCR analysis was used to check the transfection efficiency. The $TCF4^{S163A}$ cell lines were constructed by transfecting with corresponding mutating plasmids in CRC cells as mentioned above.

**12. Colony formation detection**

For the colony formation assay, $1\times10^3$ cells were seeded into 6-well plates and incubated in complete culture medium at 37°C. After 2 weeks of incubation, the colonies were fixed with 4% paraformaldehyde and stained with 0.5% crystal violet. Colonies containing at least 50 cells were scored and observed under an IX71 inverted phase-contrast fluorescence microscope (Olympus, Tokyo, Japan). The data are presented as the mean ± standard deviation of five randomly selected fields.

**13. Cell viability assay**

Cell viability was assayed by a CCK-8 reagent (HY-K0301, MedChemExpress). In brief, the cells were seeded into 96-well plates and incubated with the indicated treatments. Subsequently, 100 µl of fresh medium was added to cells containing 10 µl of CCK-8 solution and incubated for 2 h (37°C, 5% CO2). Afterward, the absorbance was measured spectrophotometrically at 450 nm. The collected values were normalized to blank wells and the relative cell viability was normalized to the respective DMSO-treated wells.

**14. Cell death assay**

For the cell death assay, the cell cultures were treated with different stimuli, collected using trypsin and without EDTA, washed twice with PBS, and resuspended in 100 µl binding buffer with 5 µl propidium iodide (PI) (556463, BD Biosciences) for 5 min. The cells were later analyzed using a CytoFLEX cytometer instrument (Beckman Coulter, Brea, CA, USA) and analyzed by CytExpert software (Beckman Coulter).

**15. MDA assay**

The relative MDA concentration in cell or tumor lysates was assessed using a Lipid Peroxidation (MDA) Assay Kit (ab118970, Abcam), according to the manufacturer's instructions. This assay measures MDA reaction with thiobarbituric acid (TBA) that

generates an MDA-TBA adduct in a sample. The MDA-TBA adduct can be quantified colorimetrically (OD = 532 nm).

**16. 4-HNE assay**

ELISA kits (Cat#MBS161454, MyBioSource, San Diego, CA, USA) were used to evaluate the concentration of 4-HNE according to the manufacturer's protocol.

**17. ELISA**

The concentrations of CCL2, CCL3, CCL13, and CSF1 were measured using ELISA kits (Cat#EK187, Cat#EK161, Cat#EK167, and Cat#EK1144, respectively; MultiSciences, Hangzhou, China), whereas CCL7, CCL8, CCL15, and CCL23 were measured using ELISA kits (Cat#MBS2024040, Cat#MBS3803299, Cat#MBS2018910, and Cat#MBS703637, respectively; MyBioSource, San Diego, CA, USA). All ELISA assays were performed according to the manufacturers' protocols.

**18. Transwell assay**

Cell migration was assessed using Transwell chambers (8 μm, 24-well inserts; Corning, Lowell, MA, USA). A total of $1\times10^4$ of THP-1 cells were seeded in the upper transwell chamber with 8μm polyester membrane and cultured in serum-free 1640. In total $1\times10^5$ of CRC cells were seeded in the lower chamber of the 24-well plate and cultured in DMEM with 10% FBS. The chambers were then incubated at 37°C for 24 hours. Non-migrated cells on the upper surface of the membrane were carefully removed using a cotton swab. Subsequently, the migrated cells on the lower surface of the membrane were fixed with 4% paraformaldehyde and transferred to a membrane stained with 0.5% crystal violet. The number of migrated cells was counted under an IX71 microscope (Olympus, Tokyo, Japan).

**19. Animal studies**

All experimental procedures were approved by the Institutional Animal Care and Use Committee (IACUC) of Sun Yat-sen University (Approval No: SYSU-IACUC-2026-002769). Four-week-old male BALB/c nude mice were purchased from GemPharmatech LLC (Nanjing, China) and randomly divided into three groups (n=5 per group): the shNC group, the shTCF4 group, and the shTCF4 + Liproxstatin-1 group. For the subcutaneous tumor model, $5 \times 10^6$ HCT116 cells were suspended in 100 μL of

PBS and injected subcutaneously into the dorsal flank of each nude mouse. Tumor volume (TV) was measured weekly using the formula: TV ($mm^3$) = length × $(width)^2$ × 0.5. The mice were euthanized four weeks after inoculation, and the tumor tissues were excised and stored at -80°C for further immunohistochemical (IHC) analysis. For the metastasis model, $5 \times 10^6$ HCT116 cells in 100 μL of PBS were injected into the tail vein of the mice to observe metastatic spread. The mice were euthanized 1.5 months later, and the livers were collected and photographed. The number of liver metastatic tumor nodules was counted for each mouse.

**20. Immunohistochemistry (IHC) analysis**

For IHC analysis, formalin-fixed, paraffin-embedded tissues were sectioned at 4 μm thickness. Then, the sections were dewaxed in xylene, rehydrated in alcohol, retrieved in EDTA buffer (pH 8.0), and blocked with peroxidase inhibitors. Specimens were incubated with normal goat serum for 30 min at room temperature to block non-specific binding. Then sections were incubated overnight at 4 °C with antibodies, rinsed with PBS, and incubated with biotinylated secondary antibodies for 30 min at room temperature, followed by color development with 3,3'-Diaminobenzidine (DAB) and hematoxylin. Finally, the Eclipse 80i microscope (Nikon) was applied for the capture of images. IHC staining was scored by the percentage of positive area and intensity as follows: 0, no staining; 1, <10% positive, moderate, or strong intensity; 2, 10–50% positive, moderate, or strong intensity; 3, >50% positive, moderate intensity; and 4, >50% positive, strong intensity. The antibodies used are listed in Supplementary Table 2.

**21. H&E staining**

Freshly procured mouse subcutaneous tumors, mouse liver metastatic tumors, and human colorectal cancer tissue specimens were immediately fixed in 4% paraformaldehyde (PFA) solution with a pH of 7.4. Following fixation, the tissues were subjected to a systematic and gradual dehydration process. Once dehydrated, the tissues were carefully embedded in paraffin wax to create paraffin-embedded blocks. From these blocks, thin sections with a uniform thickness of 4 μm were meticulously cut. These sections were then stained using the hematoxylin and eosin (H&E) staining protocol. After staining, the H&E-stained sections were placed under a light microscope

for comprehensive histological examination, enabling the visualization and analysis of tissue morphology and cellular architecture.

**22. Cytoplasmic and nuclear protein extraction**

Nuclear and cytoplasmic proteins were extracted using a commercial kit (Beyotime) in accordance with the manufacturer's instructions. Briefly, cells were harvested and suspended in Solution A containing phenylmethylsulfonyl fluoride (PMSF). The cell suspension was then incubated on ice for 10 minutes to allow for initial cell lysis. Subsequently, 10 μL of Solution B was added to the cell lysate, and the mixture was kept on ice for an additional 1 minute to facilitate further disruption of the cell membrane and release of cytoplasmic contents. Following this, the lysate was centrifuged at 15,000 rpm. The supernatant, which contained the cytoplasmic proteins, was carefully collected and set aside for further analysis. To extract nuclear proteins, the remaining pellet was resuspended in 50 μL of nuclear protein extraction reagent supplemented with PMSF. The resuspended pellet was vortexed briefly to ensure thorough mixing. Then, the sample was centrifuged at 12,000 rpm. The resulting supernatant, now enriched in nuclear proteins, was collected.

**23. Co-immunoprecipitation**

For immunoprecipitation, whole-cell extracts were lysed in NP-40 lysis buffer (Beyotime) supplemented with a protease inhibitor cocktail (Millipore) and PMSF (Beyotime). When necessary, to disrupt protein–protein interactions, the cell lysates were treated with 1% sodium dodecyl sulfate (SDS) and incubated at 90°C for 5 minutes. The cell lysates were then centrifuged at 12,000g for 15 minutes. The resulting supernatants were collected and incubated with protein A/G magnetic beads (Invitrogen) conjugated to specific antibodies or anti-FLAG M2 magnetic beads (Sigma). After overnight incubation of the magnetic beads with the lysates, the beads were washed five times using IP washing buffer. Subsequently, the bound proteins were eluted with SDS-PAGE sample buffer for further analysis. The antibodies used are listed in Supplementary Table 2.

**24. Dual-Luciferase reporter assays**

The dual-luciferase reporter assays were conducted to detect the transcriptional

regulation of TCF4 on the promoter of GPX4 and CSF1. Briefly, serial truncations of the promoter regions of human GPX4 and CSF1 were inserted upstream of the promoter of the luciferase gene in the pGL3-Basic luciferase reporter vector. Next, the constructed pGL3 plasmids were co-transfected into CRC cells. After incubating for 48 hours, the cells were harvested and lysed, followed by measuring firefly and Renilla luciferase activity using the Dual-Luciferase Reporter Assay System (Promega, Madison, WI, USA). The sequence analysis to predict the specific interacting site of TCF4 on the GPX4 or CSF1 promoter was conducted using JASPAR. Then, the mutating dual-luciferase reporter assays were performed by mutating nucleotide A to T, T to A, C to G or G to C in the predicted regions of the GPX4 or CSF1 promoter.

**25. Chromatin immunoprecipitation (ChIP)**

The DNA fragments interacting with TCF4 were examined by ChIP assays according to the manufacturer's protocol of the EZ-Magna ChIP A/G kit (Millipore). $1 \times 10^7$ CRC cells treated with the indicated TCF4 were harvested and crosslinked in 4% paraformaldehyde at room temperature for 10 min, followed by isolating the chromatins with cell lysis buffer and nuclear lysis buffer. The chromatin was further sonicated to generate short fragments between 500 bp and 700 bp and then hybridized with specific antibodies. The binding complexes were immunoprecipitated with protein A/G-coated magnetic beads at 4°C overnight. The cross-linked DNA was eluted from the beads and evaluated by qRT-PCR analysis.
