# Supplementary material for: O-GlcNAcylated TCF4 Drives Ferroptosis Resistance and Tumor-Associated Macrophage Infiltration to Promote Colorectal Cancer Liver Metastasis
Source: Cancers (Basel). 2026 Sep 24;18(19):3105. doi: 10.3390/cancers18193105 (PMC13664046; doi:10.3390/cancers18193105)
Supplement: Supplementary file 1 [file cancers-18-03105-s001.zip › Supplementary Figures.pdf]

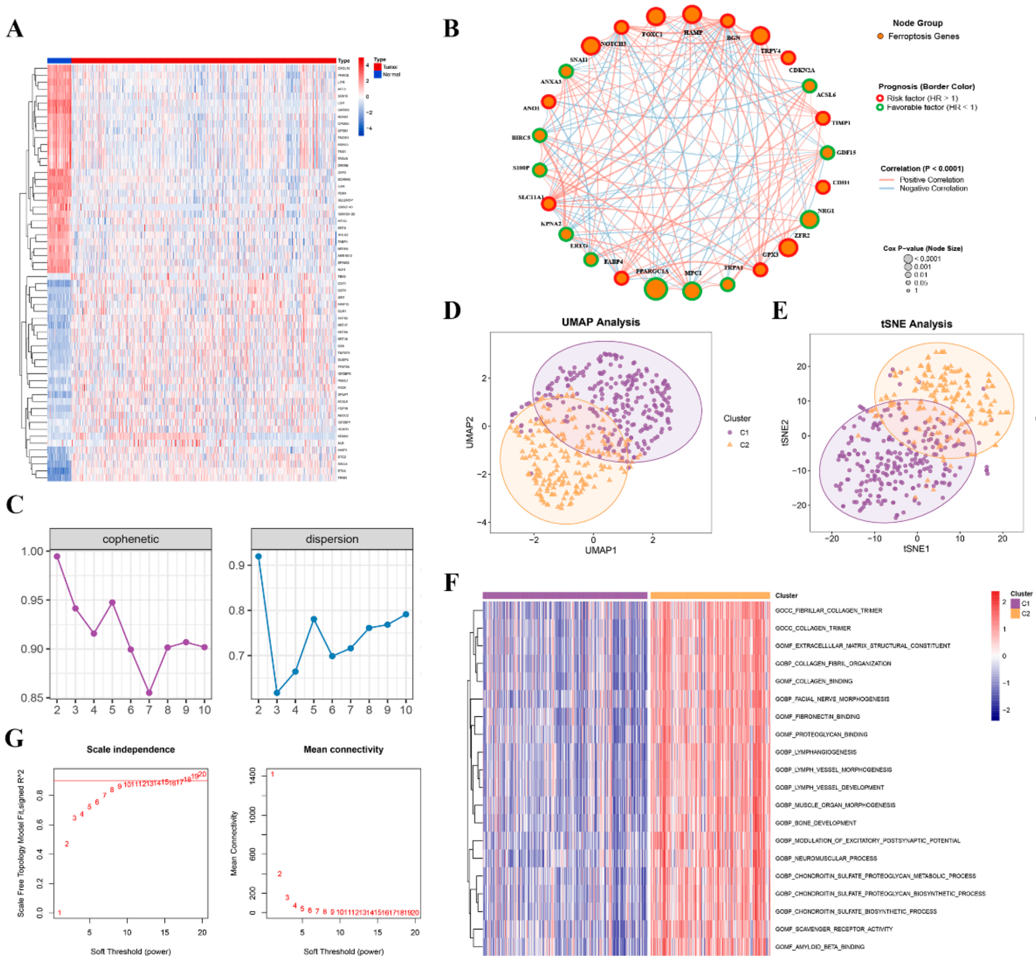

Supplementary Figure S1. Identification of ferroptosis-related genes (FRGs) and molecular subclusters in colorectal cancer based on the TCGA-COAD cohort. A. Heatmap showing differentially expressed FRGs between tumor and normal tissues in the TCGA-COAD cohort. B. Co-expression correlation network and prognostic value analysis of the 25 prognostic FRGs. C. Selection validation of the optimal number of clusters for the NMF algorithm (Cophenetic correlation and dispersion plots). D. UMAP dimensionality reduction scatter plot of the C1 and C2 subclusters. E. t-SNE dimensionality reduction scatter plot of the C1 and C2 subclusters. F. Heatmap showing GO functional enrichment analysis between the C1 and C2 subclusters. G. Scale-free topology fit index and mean connectivity analysis for determining the optimal soft threshold ($\beta = 12$) in WGCNA network construction.

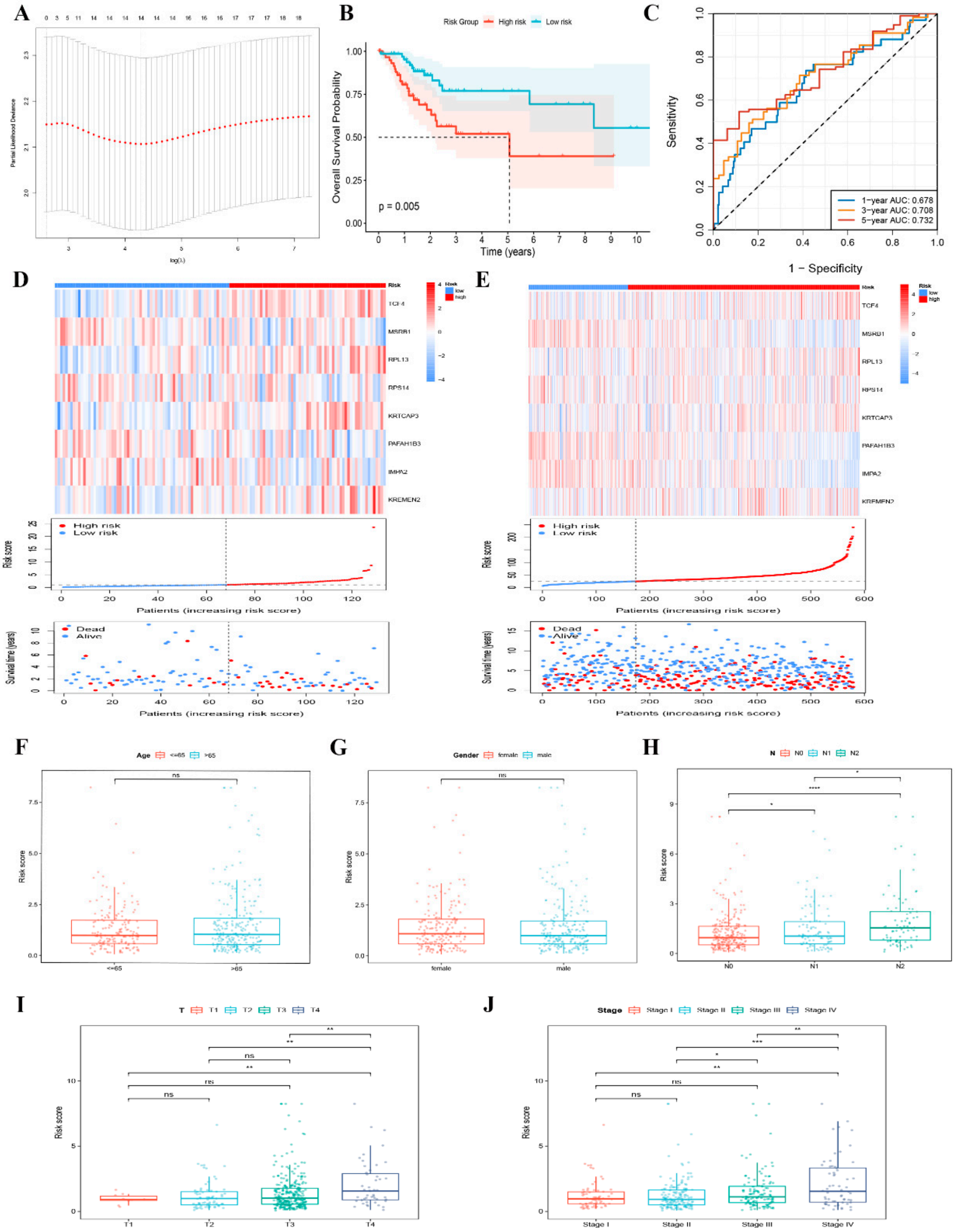
A
Partial Likelihood Deviance
log(λ)
B
Risk Group
High risk
Low risk
Overall Survival Probability
p = 0.005
Time (years)
C
Sensitivity
1 − Specificity
1−year AUC: 0.678
3−year AUC: 0.708
5−year AUC: 0.732
D
E
Risk
low
high
TCF4
MSRB1
RPL13
RPS14
KRTCAP3
PAFAH1B3
IMPA2
KREMEN2
High risk
Low risk
Risk score
Patients (increasing risk score)
Dead
Alive
Survival time (years)
F
Age
<=65
>65
Risk score
ns
G
Gender
female
male
H
N
N0
N1
N2
I
T
T1
T2
T3
T4
J
Stage
Stage I
Stage II
Stage III
Stage IV

Supplementary Figure S2. Construction and validation of a prognostic risk model based on ferroptosis-related genes in colorectal cancer. A. Cross-validation error curve and partial likelihood deviance plot in the LASSO regression model. B. Kaplan–Meier overall survival curves for high- and low-risk patients in the internal validation set. C. Time-dependent ROC curves for predicting 1-, 3-, and 5-year OS in the internal validation set. D. Distribution of risk scores, survival status, and gene expression heatmap in the internal validation set. E. Distribution of risk scores, survival status, and gene expression heatmap in the external validation set. F-J. Stratified differential analysis of risk scores across different clinical feature subgroups, including age (F), gender (G), N stage (H), T stage (I), and overall clinical stage (J). The statistical difference was assessed through a two-sided Mann–Whitney U test in (F, G) and Kruskal–Wallis test followed by Dunnett tests in (H-J). ns, P >0.05; *, $P < 0.05$; **, $P < 0.01$; ***, $P < 0.001$; ****, $P < 0.0001$.

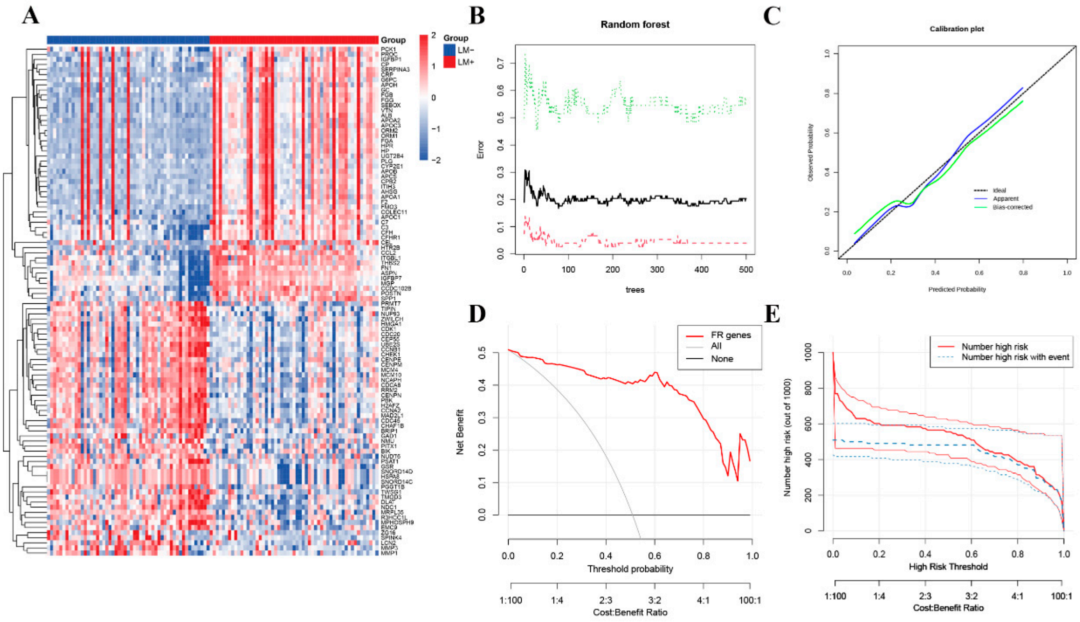

Supplementary Figure S3. Identification of liver metastasis (LM)-associated genes and construction of a diagnostic nomogram for LM in colorectal cancer. A. Heatmap of differentially expressed genes between primary CRC and CRLM samples. B. Plot of the relationship between the error rate and the number of decision trees in the RF model. C. Calibration curve of the diagnostic nomogram for CRLM. D. Decision curve analysis (DCA) evaluating the clinical net benefit of the diagnostic nomogram for CRLM. E. Clinical impact curve of the diagnostic nomogram for CRLM.

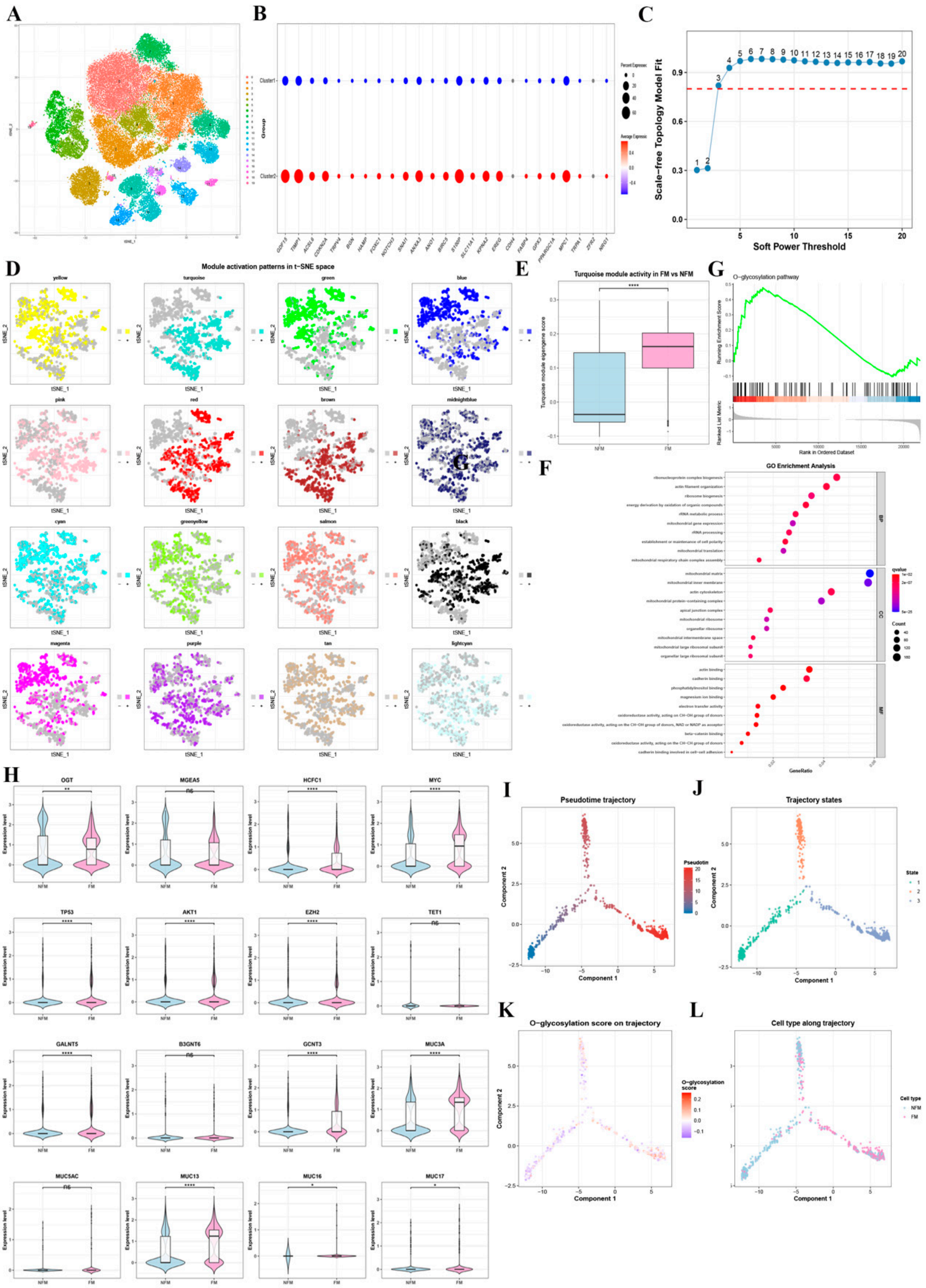
A
B
C
Scale−free Topology Model Fit
Soft Power Threshold
D
Module activation patterns in t−SNE space
yellow
turquoise
green
blue
pink
red
brown
midnightblue
cyan
greenyellow
salmon
black
magenta
purple
tan
lightcyan
tSNE_1
tSNE_2
E
Turquoise module activity in FM vs NFM
Turquoise module eigengene score
NFM
FM
G
O−glycosylation pathway
Running Enrichment Score
Ranked List Metric
Rank in Ordered Dataset
F
GO Enrichment Analysis
GeneRatio
H
OGT
MGEA5
HCFC1
MYC
TP53
AKT1
EZH2
TET1
GALNT5
B3GNT6
GCNT3
MUC3A
MUC5AC
MUC13
MUC16
MUC17
Expression level
I
Pseudotime trajectory
Component 1
Component 2
J
Trajectory states
State
K
O−glycosylation score on trajectory
O−glycosylation score
L
Cell type along trajectory
Cell type

Supplementary Figure S4. Identification of ferroptosis-associated malignant cell subclusters and characterization of their metabolic and glycosylation features through single-cell and pseudotime trajectory analyses. A. Unsupervised clustering t-SNE plot for the subdivision of cell subclusters. B. Bubble plot of differential expression levels of ferroptosis signature genes among different malignant cell subclusters (Cluster 1 and 2). C. Scale-free topology model fit distribution for determining the optimal soft threshold in hdWGCNA analysis. D. Activation pattern distribution in the t-SNE space of each co-expression module identified by hdWGCNA. E. Box plot comparing the overall activity of the turquoise module between the NFM and FM groups. F. Bubble plot of GO functional enrichment analysis of genes in the turquoise module. G. GSEA showing the significant activation of the O-glycosylation pathway in the FM group. H. Violin plots showing the differential expression levels of key metabolism- and glycosylation-related genes between the NFM and FM groups. I. Pseudotime cell trajectory distribution of malignant cells evolving toward the FM phenotype. J. Scatter plot of different branch states occupied by cell evolution in the pseudotime trajectory analysis. K. Scatter plot of the dynamic distribution of O-glycosylation scores along the entire cell pseudotime evolution trajectory. L. Distribution of NFM and FM cells along the pseudotime evolution trajectory. The statistical difference was assessed through a two-sided Mann–Whitney U test in (E, H). ns, P >0.05; *, $P < 0.05$; **, $P < 0.01$; ****, $P < 0.0001$.

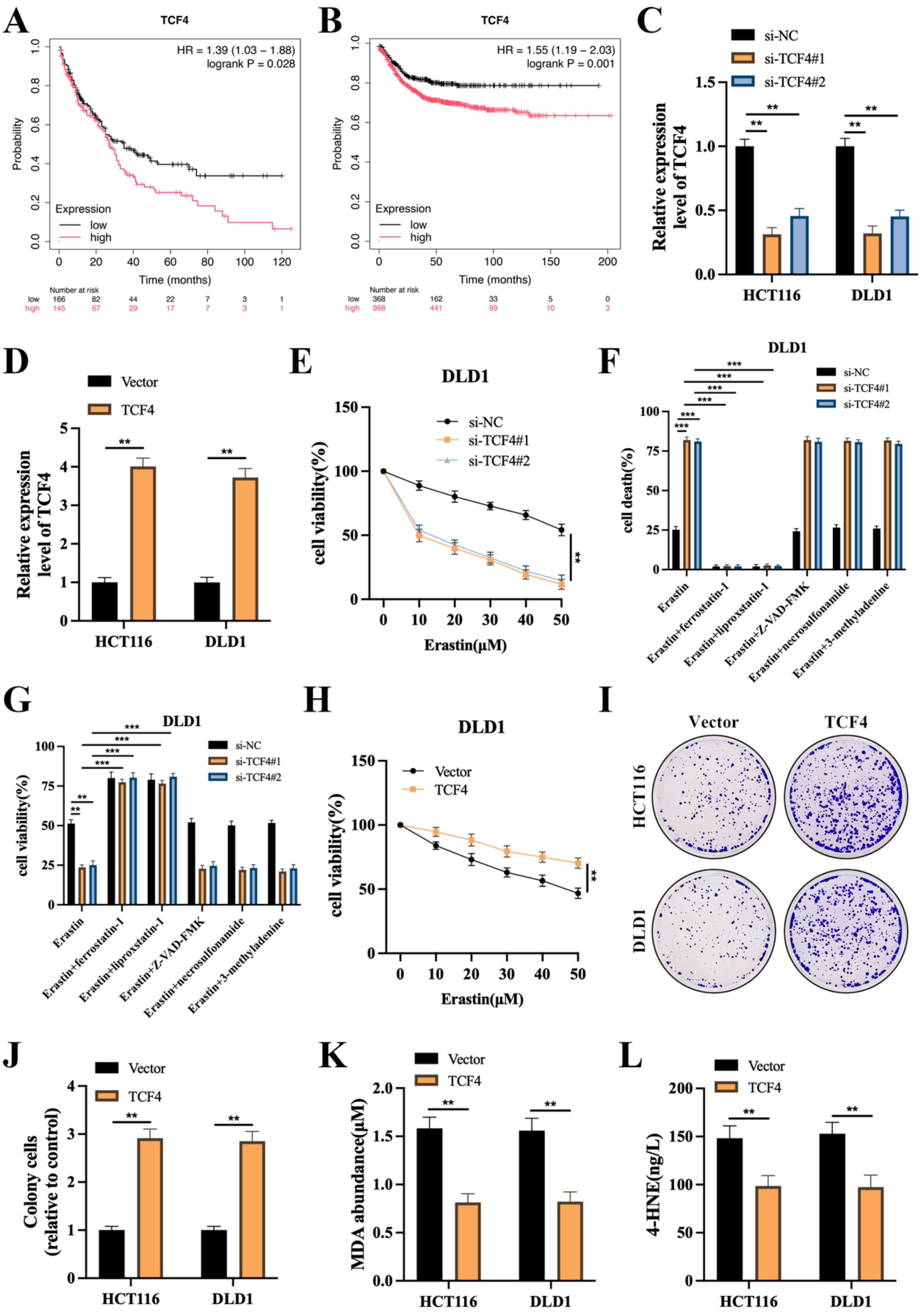
A
TCF4
HR = 1.39 (1.03 – 1.88)
logrank P = 0.028
Probability
Expression
low
high
Time (months)
Number at risk
low 166 82 44 22 7 3 1
high 145 67 29 17 7 3 1
B
TCF4
HR = 1.55 (1.19 – 2.03)
logrank P = 0.001
Probability
Expression
low
high
Time (months)
Number at risk
low 368 162 33 5 0
high 968 441 99 10 3
C
si-NC
si-TCF4#1
si-TCF4#2
Relative expression level of TCF4
HCT116
DLD1
D
Vector
TCF4
Relative expression level of TCF4
HCT116
DLD1
E
DLD1
si-NC
si-TCF4#1
si-TCF4#2
cell viability(%)
Erastin(μM)
F
DLD1
si-NC
si-TCF4#1
si-TCF4#2
cell death(%)
Erastin
Erastin+ferrostatin-1
Erastin+liproxstatin-1
Erastin+Z-VAD-FMK
Erastin+necrosulfonamide
Erastin+3-methyladenine
G
DLD1
si-NC
si-TCF4#1
si-TCF4#2
cell viability(%)
Erastin
Erastin+ferrostatin-1
Erastin+liproxstatin-1
Erastin+Z-VAD-FMK
Erastin+necrosulfonamide
Erastin+3-methyladenine
H
DLD1
Vector
TCF4
cell viability(%)
Erastin(μM)
I
Vector
TCF4
HCT116
DLD1
J
Vector
TCF4
Colony cells (relative to control)
HCT116
DLD1
K
Vector
TCF4
MDA abundance(μM)
HCT116
DLD1
L
Vector
TCF4
4-HNE(ng/L)
HCT116
DLD1

Supplementary Figure S5. TCF4 regulates ferroptosis sensitivity in colorectal cancer cells. A. The recurrence-free survival for CRC patients was analyzed using Kaplan–Meier curves (log-rank test). B. The post-progression survival for CRC patients was analyzed using Kaplan–Meier curves (log-rank test). C. The silencing of TCF4 was confirmed using qRT-PCR analysis (n = 3). D. The TCF4 overexpression vector was confirmed using qRT-PCR analysis (n = 3). E. Cell viability of DLD1 expressing si-NC or si-TCF4 treated with different concentrations of erastin for 24 h (n = 3). F. Cell death measurement of DLD1 expressing si-NC or si-TCF4 treated with erastin (20 μM) in the absence or presence of ferrostatin-1 (2 μM), liproxstatin-1 (1 μM), Z-VAD-FMK (10 μM), necrosulfonamide (0.5 μM), or 3-methyladenine (250 μM) for 24 h (n = 3). G. Cell viability measurement of DLD1 expressing si-NC or si-TCF4 treated with erastin (50 μM) in the absence or presence of ferrostatin-1 (2 μM), liproxstatin-1 (1 μM), Z-VAD-FMK (10 μM), necrosulfonamide (0.5 μM), or 3-methyladenine (250 μM) for 24 h (n = 3). H. Cell viability of DLD1 expressing Vector or TCF4 treated with different concentrations of erastin for 24 h (n = 3). I-J. Colony formation of CRC cells expressing Vector or TCF4 (n = 3). K. MDA production in CRC cells expressing Vector or TCF4 (n = 3). L. 4-HNE production in CRC cells expressing Vector or TCF4 (n = 3). The statistical difference was assessed through one-way ANOVA followed by Dunnett tests in (C, E-G) and unpaired two-sided Student's t-test in (D, H, J-L). Error bars show the SD from three independent experiments. **, $P < 0.01$; ***, $P < 0.001$.

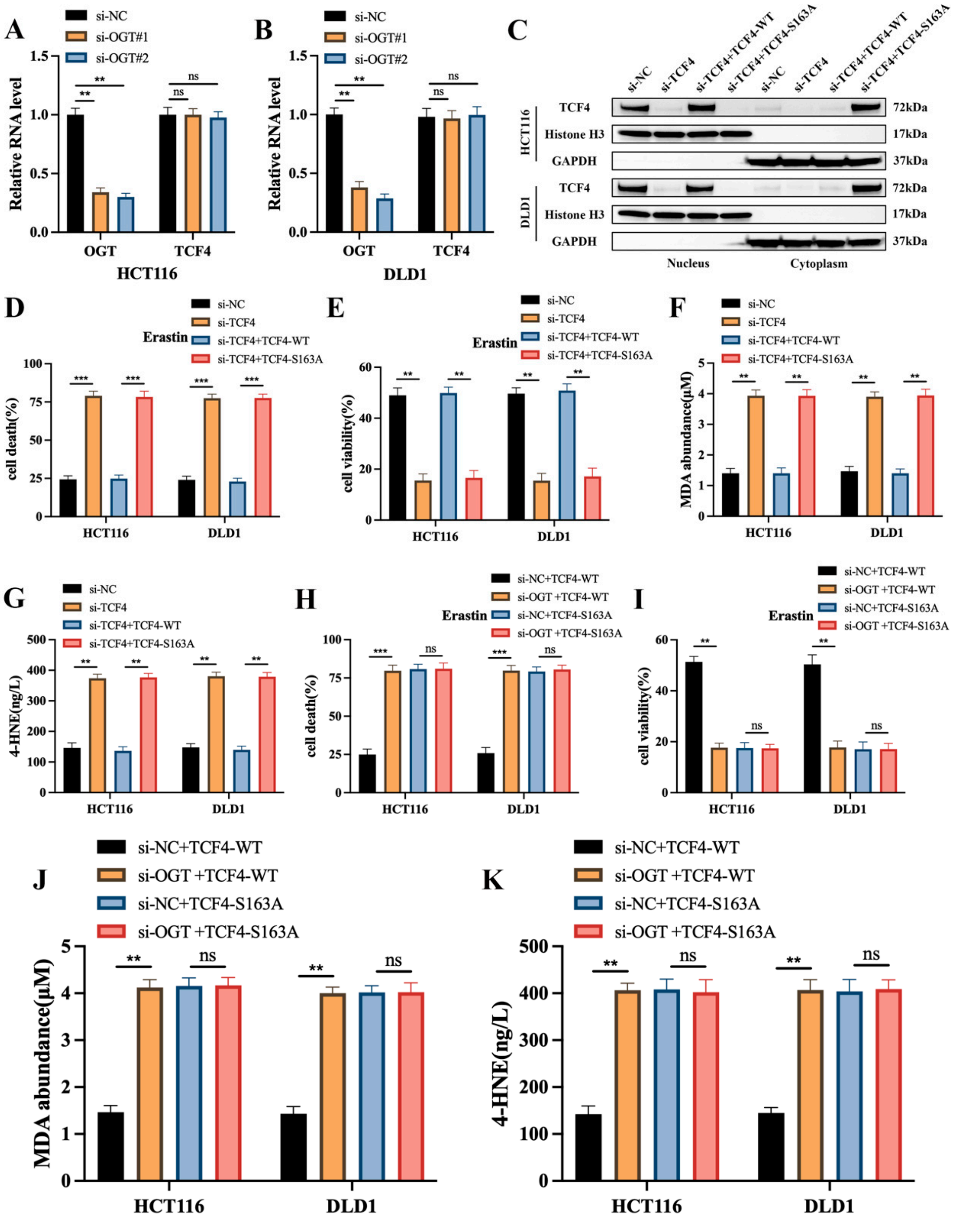
A
B
C
D
E
F
G
H
I
J
K
HCT116
DLD1
si-NC
si-OGT#1
si-OGT#2
Relative RNA level
OGT
TCF4
si-TCF4
si-TCF4+TCF4-WT
si-TCF4+TCF4-S163A
Histone H3
GAPDH
72kDa
17kDa
37kDa
Nucleus
Cytoplasm
Erastin
cell death(%)
cell viability(%)
MDA abundance(μM)
4-HNE(ng/L)
si-NC+TCF4-WT
si-OGT +TCF4-WT
si-NC+TCF4-S163A
si-OGT +TCF4-S163A

Supplementary Figure S6. OGT-mediated O-GlcNAcylation of TCF4 at Ser163 regulates ferroptosis in colorectal cancer cells. A-B. OGT silencing was confirmed using qRT-PCR analysis (n = 3). C. Western blot analyses were performed to examine the nuclear and cytoplasmic localization of TCF4 in CRC cells under different treatment conditions (n = 3). D-G. Ferroptotic events were evaluated in CRC cells transfected with the indicated plasmids: cell death (D), cell viability (E), MDA levels (F), and 4-HNE levels (G) (n = 3). H-K. Ferroptotic events were evaluated in CRC cells transfected with the indicated plasmids: cell death (H), cell viability (I), MDA levels (J), and 4-HNE levels (K) (n = 3). The statistical difference was assessed through one-way ANOVA followed by Dunnett tests in (A, B) and unpaired two-sided Student's t-test in (D-K). Error bars show the SD from three independent experiments. ns, $P > 0.05$; **, $P < 0.01$; ***, $P < 0.001$.

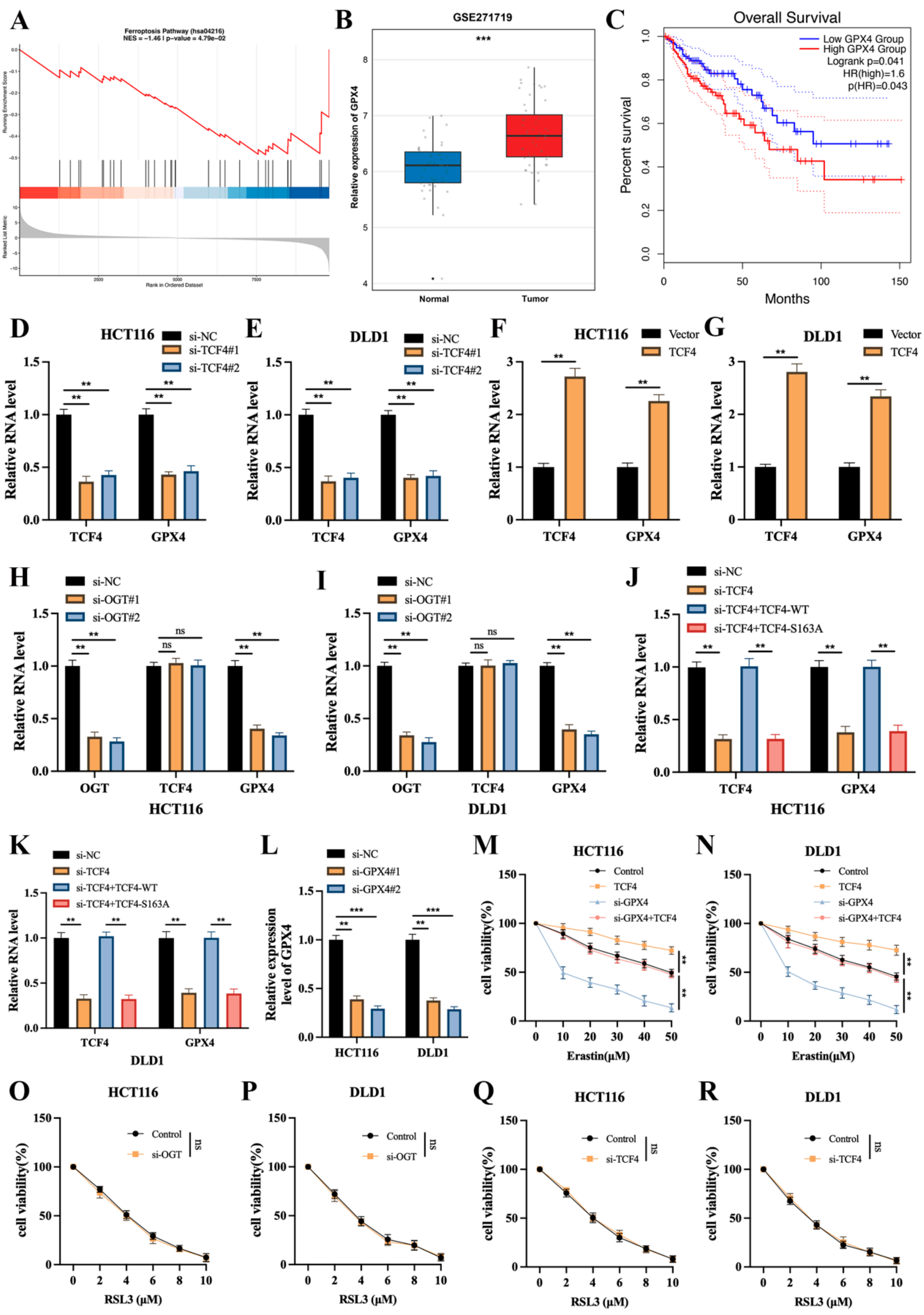

A
Ferroptosis Pathway (hsa04216)
NES = -1.46 | p-value = 4.79e-02
Running Enrichment Score
Ranked List Metric
Rank in Ordered Dataset
B
GSE271719
***
Relative expression of GPX4
Normal
Tumor
C
Overall Survival
Low GPX4 Group
High GPX4 Group
Logrank p=0.041
HR(high)=1.6
p(HR)=0.043
Percent survival
Months
D
HCT116
si-NC
si-TCF4#1
si-TCF4#2
Relative RNA level
TCF4
GPX4
E
DLD1
si-NC
si-TCF4#1
si-TCF4#2
Relative RNA level
TCF4
GPX4
F
HCT116
Vector
TCF4
Relative RNA level
TCF4
GPX4
G
DLD1
Vector
TCF4
Relative RNA level
TCF4
GPX4
H
si-NC
si-OGT#1
si-OGT#2
Relative RNA level
OGT
TCF4
GPX4
HCT116
I
si-NC
si-OGT#1
si-OGT#2
Relative RNA level
OGT
TCF4
GPX4
DLD1
J
si-NC
si-TCF4
si-TCF4+TCF4-WT
si-TCF4+TCF4-S163A
Relative RNA level
TCF4
GPX4
HCT116
K
si-NC
si-TCF4
si-TCF4+TCF4-WT
si-TCF4+TCF4-S163A
Relative RNA level
TCF4
GPX4
DLD1
L
si-NC
si-GPX4#1
si-GPX4#2
Relative expression level of GPX4
HCT116
DLD1
M
HCT116
Control
TCF4
si-GPX4
si-GPX4+TCF4
cell viability(%)
Erastin(μM)
N
DLD1
Control
TCF4
si-GPX4
si-GPX4+TCF4
cell viability(%)
Erastin(μM)
O
HCT116
Control
si-OGT
ns
cell viability(%)
RSL3 (μM)
P
DLD1
Control
si-OGT
ns
cell viability(%)
RSL3 (μM)
Q
HCT116
Control
si-TCF4
ns
cell viability(%)
RSL3 (μM)
R
DLD1
Control
si-TCF4
ns
cell viability(%)
RSL3 (μM)

Supplementary Figure S7. O-GlcNAcylation and TCF4 regulate GPX4 expression and ferroptosis sensitivity in colorectal cancer cells. A. GSEA enrichment analysis of the number of pathways at the mRNA level. B. GPX4 expression data from the GEO database for colorectal cancer were analysed (n = 6). C. The overall survival for CRC patients was analyzed using Kaplan–Meier curves (log-rank test). D-G. Relative TCF4 and GPX4 expressions were detected in HCT116 and DLD1 cells using qRT-PCR (n = 3). H-I. Relative OGT, TCF4 and GPX4 expressions were detected in HCT116 and DLD1 cells using qRT-PCR (n = 3). J-K. Relative TCF4 and GPX4 expressions were detected in HCT116 and DLD1 cells using qRT-PCR (n = 3). L. The silencing of GPX4 was confirmed using qRT-PCR analysis (n = 3). M-N. Cell viability of CRC cells expressing TCF4 or si-GPX4 treated with different concentrations of erastin for 24 h (n = 3). O-P. Cell viability of CRC cells expressing si-NC or si-OGT treated with different concentrations of RSL3 for 24 h (n = 3). Q-R. Cell viability of CRC cells expressing si-NC or si-TCF4 treated with different concentrations of RSL3 for 24 h (n = 3). The statistical difference was assessed through a two-sided Mann–Whitney U test in (B); one-way ANOVA followed by Dunnett tests in (D, E, H, I, L-N); and an unpaired two-sided Student's t-test in (F, G, J, K, O-R). Error bars show the SD from three independent experiments. ns, $P > 0.05$; **, $P < 0.01$; ***, $P < 0.001$.

A

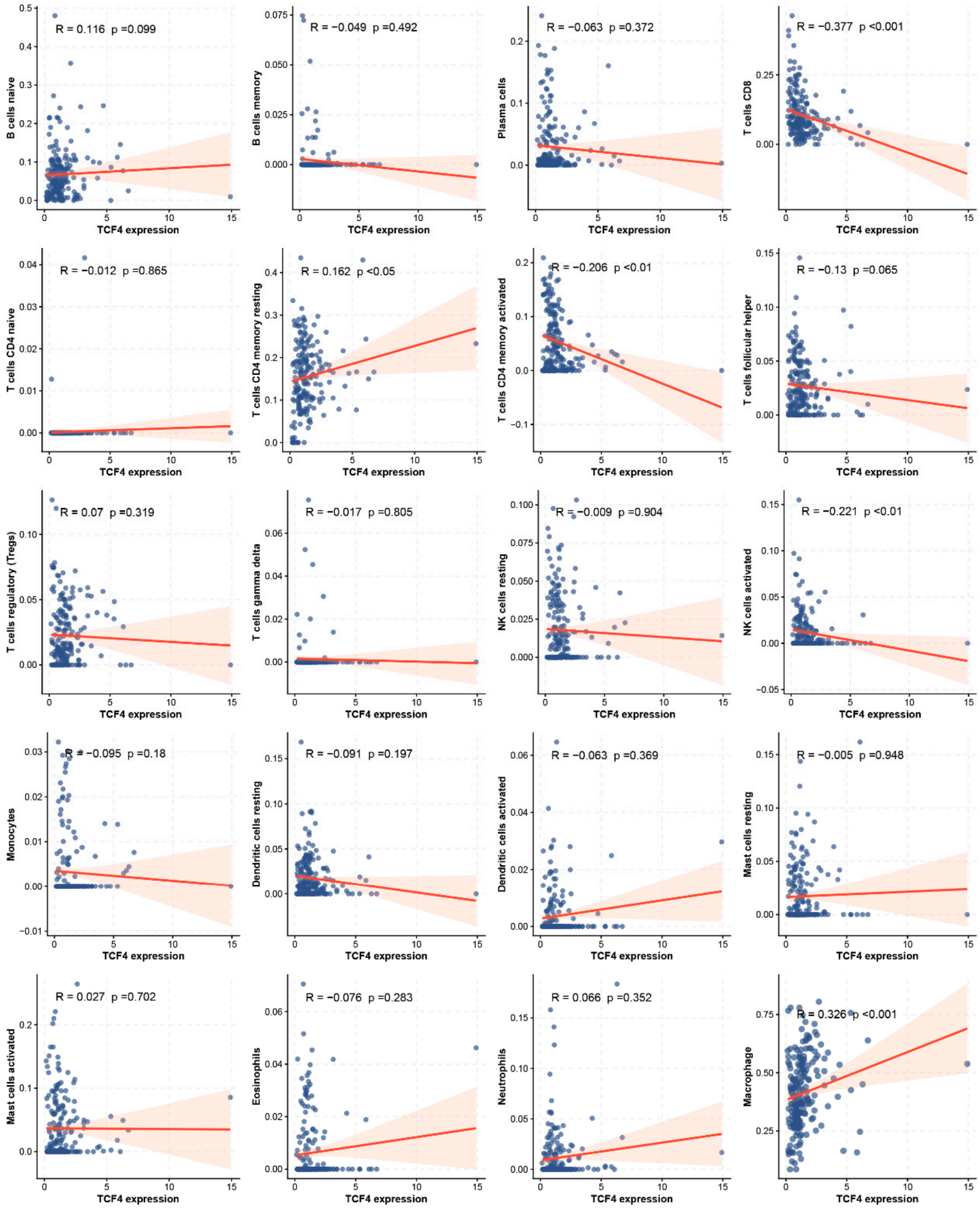

Supplementary Figure S8. Correlation between TCF4 expression and immune cell infiltration in colorectal cancer. A. Scatter plot and linear fitting curves showing the Spearman correlation analysis between TCF4 expression levels and the infiltration abundance of immune cells in the TCGA-COAD cohort.

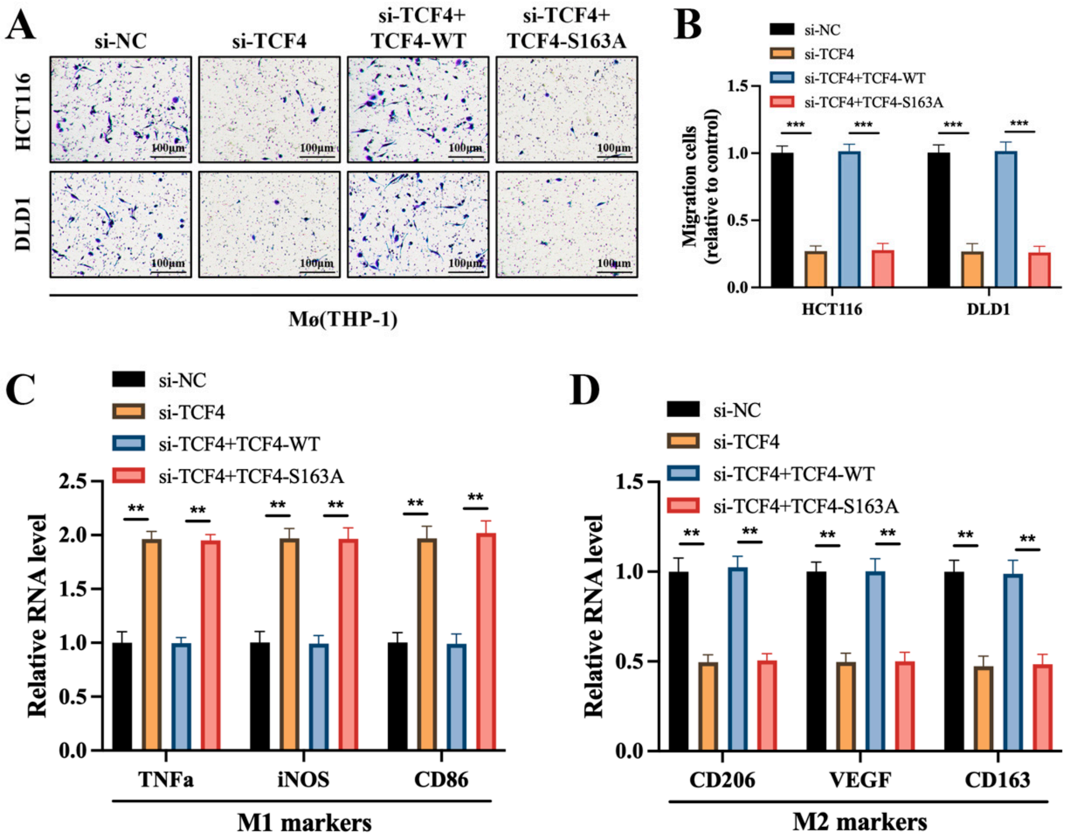

Supplementary Figure S9. O-GlcNAcylation of TCF4 at Ser163 in CRC cells promotes macrophage M2 polarization in co-culture systems. A-B. Transwell migration assay of THP-1 cells cocultured with the indicated CRC cells (n = 3). Scale bar, 100 μm. C. Relative mRNA expression of TNFa, iNOS, CD86 in THP-1 cells (n = 3). D. Relative mRNA expression of CD206, VEGF, CD163 in THP-1 cells (n = 3). The statistical difference was assessed through unpaired two-sided Student's t-test in (B-D). Error bars show the SD from three independent experiments. **, $P < 0.01$; ***, $P < 0.001$.

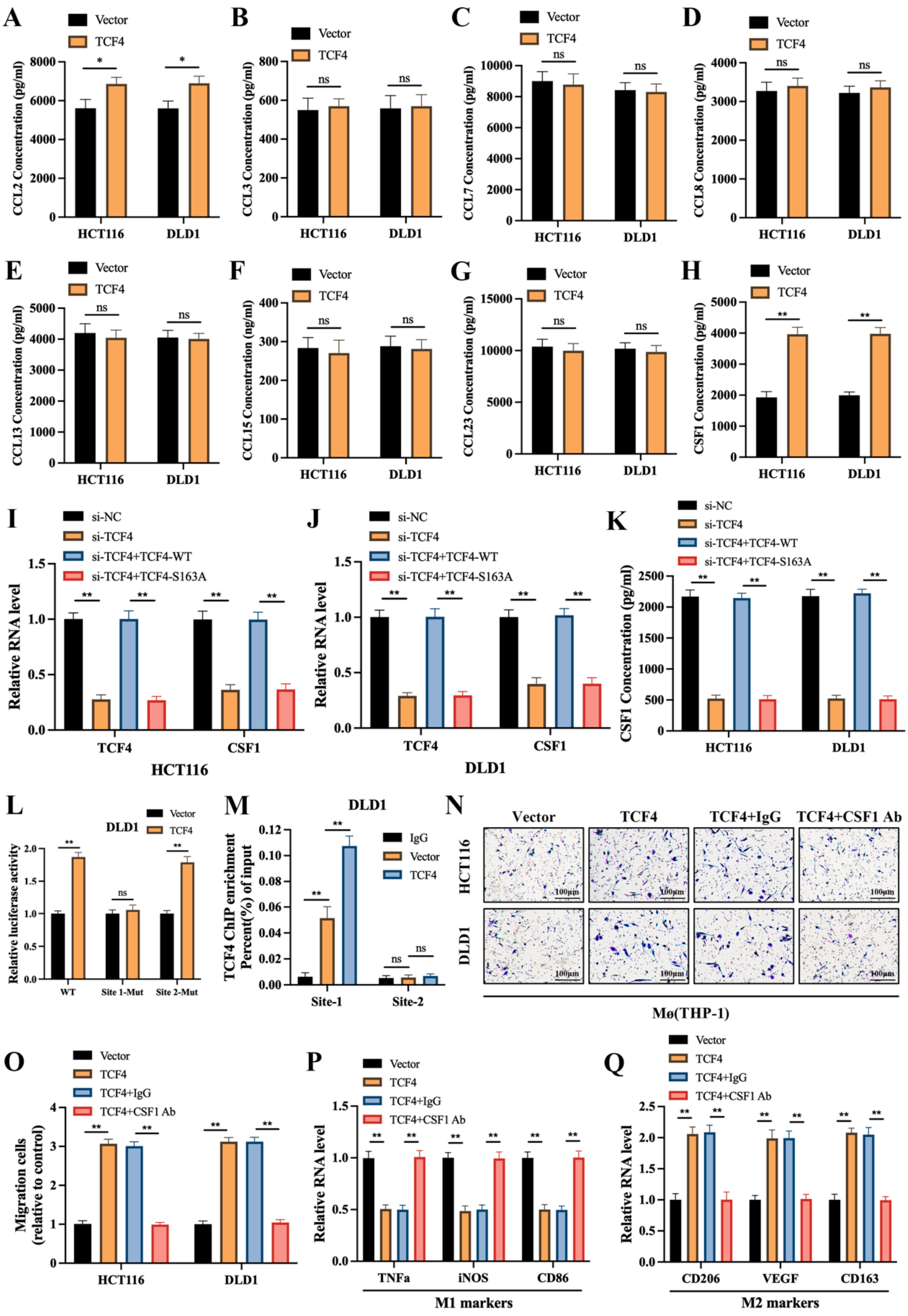

A
B
C
D
E
F
G
H
I
J
K
L
M
N
O
P
Q
Vector
TCF4
CCL2 Concentration (pg/ml)
CCL3 Concentration (pg/ml)
CCL7 Concentration (pg/ml)
CCL8 Concentration (pg/ml)
CCL13 Concentration (pg/ml)
CCL15 Concentration (ng/ml)
CCL23 Concentration (pg/ml)
CSF1 Concentration (pg/ml)
HCT116
DLD1
si-NC
si-TCF4
si-TCF4+TCF4-WT
si-TCF4+TCF4-S163A
Relative RNA level
TCF4
CSF1
Relative luciferase activity
WT
Site 1-Mut
Site 2-Mut
IgG
TCF4 ChIP enrichment Percent(%) of input
Site-1
Site-2
TCF4+IgG
TCF4+CSF1 Ab
Mø(THP-1)
Migration cells (relative to control)
TNFa
iNOS
CD86
M1 markers
CD206
VEGF
CD163
M2 markers

Supplementary Figure S10. O-GlcNAcylation of TCF4 at Ser163 promotes CSF1 expression and macrophage M2 polarization in colorectal cancer cells. A-H. Concentrations of CCL2, CCL3, CCL7, CCL8, CCL13, CCL15, CCL23, and CSF1 in CRC cells expressing Vector or TCF4 (n = 3). I-J. Relative TCF4 and CSF1 expressions were detected in HCT116 and DLD1 cells using qRT-PCR (n = 3). K. CSF1 concentrations in TCF4-knockdown CRC cells reconstituted with TCF4-WT or TCF4-S163A (n = 3). L. Relative activities of the WT and Mut luciferase reporters (n = 3). M. ChIP-qPCR analysis of TCF4-enriched chromatin in DLD1 cells (n = 3). N-O. Transwell migration assay of THP-1 cells cocultured with indicated CRC cells (n = 3). Scale bar, 100 μm. P. Relative mRNA expression of TNFa, iNOS, CD86 in THP-1 cells (n = 3). Q. Relative mRNA expression of CD206, VEGF, CD163 in THP-1 cells (n = 3). The statistical difference was assessed through an unpaired two-sided Student's t-test in (A-L, O-Q) and one-way ANOVA followed by Dunnett's test in (M). Error bars show the SD from three independent experiments. ns, $P > 0.05$; *, $P < 0.05$; **, $P < 0.01$.
