# Supplementary material for: O-GlcNAcylated TCF4 Drives Ferroptosis Resistance and Tumor-Associated Macrophage Infiltration to Promote Colorectal Cancer Liver Metastasis
Source: Cancers (Basel). 2026 Sep 24;18(19):3105. doi: 10.3390/cancers18193105 (PMC13664046; doi:10.3390/cancers18193105)
Supplement: Supplementary file 1 [file cancers-18-03105-s001.zip › Supplementary Tables.pdf]

Table S1. Primers and siRNAs used in the experiments.

Target gene	Sequence (5'-3')	Application
TCF4	F: CCGATGACGAGGGTGATGAG<br>R: CCTTCTGCTCTGGTGTCAGG	qRT-PCR
GPX4	F: GGGCACATGGTTAACCTGGA<br>R: TTCCCGAACTGGTTACACGG	qRT-PCR
TNFa	F: CTCTGTATCCGTGGCGGAC<br>R: GATGACTTGAAGCCCCACTAGC	qRT-PCR
iNOS	F: GAACGACTCCAACACTATGACTT<br>R: CCCTAAAATTGAACGTCCAGGT	qRT-PCR
CD86	F: CCATTGGCACATATCTACTCGTC<br>R: GTGTCTTGACATGCTGCAAAGG	qRT-PCR
CD206	F: GTTCTGTGCATTAGGTGAAACG<br>R: GTAAACGGTCGCTGTATTGTC	qRT-PCR
VEGF	F: GAAGGAGGTCGAGTCGGAA<br>R: CGCTCAGAGTTAGTACGAGG	qRT-PCR
CD163	F: CACTTCCCTGAGTTCAACTGTTT<br>R: CACTTTTGTTCACATCGCCCT	qRT-PCR
CSF1	F: ACCCCCTCTACCATCACCTC<br>R: CACAGCAGCACAAAGGAACC	qRT-PCR
OGT	F: GTTTGCTGCTGCCCATTCAA<br>R: CGCGTATAACACTGCAAGGC	qRT-PCR
GAPDH	F: CAAATTCCATGGCACCGTCA<br>R: ATGATGTTCTGGAGAGCCCC	qRT-PCR
GPX4-Site1	F: TGGAGCTGTGATGAGAAGCA<br>R: CCAGGCTGATCTCGAACTCC	ChIP
GPX4-Site2	F: TTTTCTGAGCACATACCGCG<br>R: GGCACTGAGACCTGAGAGTC	ChIP
CSF1-Site1	F: AGGCTTAGGCTCATCCTACT<br>R: GCTGGTACCCATGCTCTCTC	ChIP
CSF1-Site2	F: GATCCAGAGGTGACTGAGCA<br>R: TGTTCCCATTGGACTTGGCT	ChIP
si-TCF4#1	sense: UAAACGAUCUUCAAUUCGGTT<br>antisense: CCGAAUUGAAGAUCGUUUATT	si-RNA
si-TCF4#2	sense: AUUUGAUAUCCUUCUUGUCTT<br>antisense: GACAAGAAGGAUAUCAAAUTT	si-RNA
si-GPX4#1	sense: UUUGAUCUCUUCGUUACUCTT<br>antisense: GAGUAACGAAGAGAUCAAAGTT	si-RNA
si-GPX4#2	sense: AUCGAAUUUGACGUUGUAGTT<br>antisense: CUACAACGUCAAAUUCGAUTT	si-RNA
si-OGT#1	sense: UAAUGCUUUACGAUACAAGTT<br>antisense: CUUGUAUCGUAAAGCAUUATT	si-RNA
si-OGT#2	sense: UUCUAAUGCUUUACGAUACTT<br>antisense: GUAUCGUAAAGCAUUAGAATT	si-RNA

Table S2. Antibodies used in the experiments.

Product	Source	Catalog no.
**Primary antibody**		
Western blotting		
anti-O-GlcNAc	Proteintech	65292-1-Ig
anti-TCF4	Abcam	ab217668
anti-Histone H3	Abcam	ab1791
anti-GAPDH	Cell Signaling Technology	2118S
Immunohistochemistry		
anti-TCF4	Abcam	ab217668
anti-4-HNE	Abcam	ab48506

anti-Ki-67	Cell Signaling Technology	9027S
anti-CD68	Abcam	ab303565
anti-F4/80	Abcam	ab300421
anti-CD206	Abcam	ab64693
anti-CD86	Abcam	ab220188
anti-CD86	Abcam	ab305361
anti-CSF1	Abcam	ab99178
anti-CSF1R	Abcam	ab315490
ChIP		
anti-TCF4	Abcam	ab217668
Immunoprecipitation		
anti-TCF4	Proteintech	22337-1-AP
**Secondary antibody:**		
Western blotting		
anti-rabbit IgG-HRP	Cell Signaling Technology	7074S
anti-mouse IgG-HRP	Cell Signaling Technology	7076S
Immunohistochemistry		
anti-rabbit IgG-HRP	Proteintech	SA00001-2
anti-mouse IgG-HRP	Proteintech	SA00001-1
